# GLS1‐Mediated Redundancy in Glutamate Accelerates Arterial Calcification via Activating NMDAR/Ca^2+^/*β*‐Catenin Pathway

**DOI:** 10.1002/advs.202414252

**Published:** 2025-04-28

**Authors:** Ziting Zhou, Bing Dong, Dayu He, Jianshuai Ma, Yun Kong, Huijin Zhu, Chen Xie, Tiecheng Yang, Xin Zhen, Zhengzhipeng Zhang, Zhaohui He, Jinkun Cheng, Aoran Huang, Jie Chen, Ruo Wu, Huiyong Yin, Yanlian Chen, Jun Tao, Hui Huang

**Affiliations:** ^1^ Department of Cardiology Joint Laboratory of Guangdong‐Hong Kong‐Macao Universities for Nutritional Metabolism and Precise Prevention and Control of Major Chronic Diseases the Eighth Affiliated Hospital of Sun Yat‐sen University Shenzhen Guangdong 518033 China; ^2^ Medical Research Center The Eighth Affiliated Hospital Sun Yat‐sen University Shenzhen Guangdong 518033 China; ^3^ Department of Nephrology The Eighth Affiliated Hospital Sun Yat‐sen University Shenzhen Guangdong 518033 China; ^4^ Department of Urology The Eighth Affiliated Hospital Sun Yat‐sen University Shenzhen Guangdong 518033 China; ^5^ Department of Oncology Sun Yat‐Sen Memorial Hospital Sun Yat‐sen University Guangzhou Guangdong 510120 China; ^6^ Department of Biomedical Sciences City University of Hong Kong Hong Kong SAR 999077 China; ^7^ Department of Hypertension and Vascular Disease The First Affiliated Hospital of Sun Yat‐sen University Guangzhou Guangdong 510080 China

**Keywords:** arterial calcification, GLS1, glutamate, NMDAR, osteogenic reprogramming, *β*‐Catenin

## Abstract

Arterial calcification is a powerful predictor of both the events and mortality associated with cardiovascular diseases in chronic kidney disease (CKD) patients. GLS1 (glutaminase 1), a rate‐limiting enzyme catalyzing the conversion of glutamine to glutamate, is disordered in various cardiovascular diseases. However, the potential interplay between GLS1‐mediated glutamate production and arterial calcification remains poorly understood. Here, LC‐MS/MS analysis of CKD patients’ samples shows an abnormally elevated activity of GLS1, reflected by the increased glutamate/glutamine ratio. Moreover, GLS1 activity is positively correlated with arterial calcification progression, and its expression is upregulated in calcified arteries. Treatment with GLS1 inhibitors or knockdown of GLS1 alleviates osteogenic reprogramming. In contrast, glutamate administration boosts the development of arterial calcification. Mechanistically, GLS1 redundancy‐regulated glutamate superfluity stimulates the activation of N‐methyl‐d‐aspartate receptors (NMDAR), leading to Ca^2+^ influx and extracellular regulated protein kinases (ERK) phosphorylation, followed by the nuclear translocation of *β*‐Catenin and acceleration of osteogenic reprogramming of vascular smooth muscle cells (VSMCs) in further. This research defines GLS1 as a key contributor to arterial calcification. Glutamate, a major product of GLS1‐mediated glutamine metabolism, exerts a deleterious effect on arterial calcification by activating NMDAR and subsequently triggering Ca^2+^ influx, which in turn exacerbates *β*‐Catenin‐regulated osteogenic reprogramming in VSMCs.

## Graphical abstract

GLS1 is a novel contributor to arterial calcification. GLS1‐catalyzed glutamate exerts the promoting effects on osteogenic reprogramming in arteries. NMDAR, a glutamate receptor, is also activated and overexpressed during arterial calcification. GLS1‐mediated redundancy in glutamate promotes calcium influx and subsequent ERK phosphorylation by activating NMDAR, leading to nuclear translocation of *β*‐catenin and thus progression of arterial calcification.

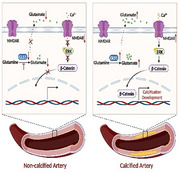

## 1 Introduction

Arterial calcification, defined as the ectopic deposition of calcium and phosphate crystals mainly composed of hydroxyapatite in arteries, is highly prevalent in patients with chronic kidney disease (CKD) and contributes to adverse clinical events.^[^ ^1a,b^ ^]^ Cardiovascular disease is the main cause of death in CKD patients.^[^ ^2^ ^]^ Arterial calcification consequently remains an increased risk of all‐cause death and adverse cardiovascular outcomes, beyond traditional risk factors.^[^ ^3a,b^ ^]^ Owing to a lack of systematic understanding of the pathological mechanisms, there is no clear and efficient anti‐calcifying strategy.

Previous studies have linked arterial calcification to metabolic disorders,^[^ ^4a,b,c^ ^]^ especially abnormal amino acid metabolism.^[^ ^4^, ^5^ ^]^ There has been a reported connection between CKD and altered glutamine metabolism. Studies demonstrated that CKD was related to higher levels of glutamine and glutamate in rat blood, possibly owing to disturbance nitrogen utilization and accelerated protein breakdown.^[^ ^6a,b,c^ ^]^ The deamidation of glutamine to glutamate is catalyzed by glutaminase1 (GLS1). The metabolic process is called glutaminolysis, in which GLS1 serves as a rate‐limiting enzyme.^[^ ^7a,b,c^ ^]^ The glutamate/glutamine ratio is widely used to reflect the activity of GLS1.^[^ ^8^ ^]^ Multiple studies have shown that GLS1 dysregulation participates in the pathogenesis of age‐associated disorders,^[^ ^9^ ^]^ pulmonary arterial hypertension (PAH),^[^ ^10^ ^]^ vascular stiffness,^[^ ^11^ ^]^ heart failure,^[^ ^12^ ^]^ and calcific aortic valve disease.^[^ ^13^ ^]^ In addition, GLS1 abnormality was also associated with the process of osteogenic differentiation.^[^ ^14a,b^ ^]^ However, little is known about whether GLS1 has participated in the process of arterial calcification.

As a pleiotropic molecular, glutamate binds to its receptors, including ionotropic receptors AMPAR (α‐amino‐3‐hydroxy‐5‐methyl‐4‐isoxazole‐propionate receptors), NMDAR (N‐methyl‐d‐aspartate receptors) and metabotropic receptors (metabotropic glutamate receptor, mGlu1‐8) to exert functions.^[^ ^15^ ^]^ NMDAR is a glutamate‐gated, voltage‐dependent, heterotetrameric transmembrane channel, with subunits classically consisting of two GluN1 and two GluN2A or GluN2B.^[^ ^16^ ^]^ Glutamate‐NMDAR axis participates in bone resorption,^[^ ^17^ ^]^ and NMDAR activation is necessary for bone formation,^[^ ^18^ ^]^ which indicates that the glutamate‐NMDAR axis may be a vital regulator of osteogenic reprogramming. Because of the similar mechanisms of bone and vascular mineralization, including the increased expression of critical osteogenic markers such as Runt‐related transcription factor 2 (RUNX2), bone morphogenetic protein 2 (BMP2) and osteocalcin (OCN),^[^ ^19a,b^ ^]^ we assumed that GLS1 and glutamate may be involved in the pathological process of arterial calcification.

Wnt/*β*‐Catenin signaling has been widely studied for its involvement in arterial calcification. Stimulated by various factors, unphosphorylated *β*‐Catenin (an active form of *β*‐Catenin) accumulate in the cytoplasm and translocate to the nucleus.^[^ ^20a,b^ ^]^ Nuclear *β*‐Catenin binds to T‐cell factor/lymphoid enhancer‐binding factor (TCF/LEF), functioning as a coactivator to drive the transcription of master osteogenic regulators, including RUNX2^[^ ^21^ ^]^ and msh homeobox 2 (MSX2),^[^ ^22^ ^]^ and thus promoting arterial calcification. In this study, we investigated the detrimental role of GLS1 in arterial calcification. Using clinical samples from donors, we verified that GLS1 was increased in calcified arteries, and GLS1‐mediated metabolism was abnormally elevated in CKD patients. We identified that GLS1 reduction impeded VSMCs osteogenic reprogramming and subsequent arterial calcification, whereas glutamate supplementation exhibited the converse effect, accelerating these processes. Mechanistically, stimulation of NMDAR by glutamate‐mediated Ca^2+^ influx and subsequent extracellular regulated protein kinases (ERK) activation, which promoted osteogenic reprogramming of VSMCs in a *β*‐Catenin dependent manner, ultimately leading to arterial calcification. These findings emphasized the harmful roles of GLS1 and glutamate in arterial calcification.

## 2 Results

### 2.1 GLS1 and Its Catalyzed Production Glutamate Are Positively Associated with Arterial Calcification

**Figure 1:**
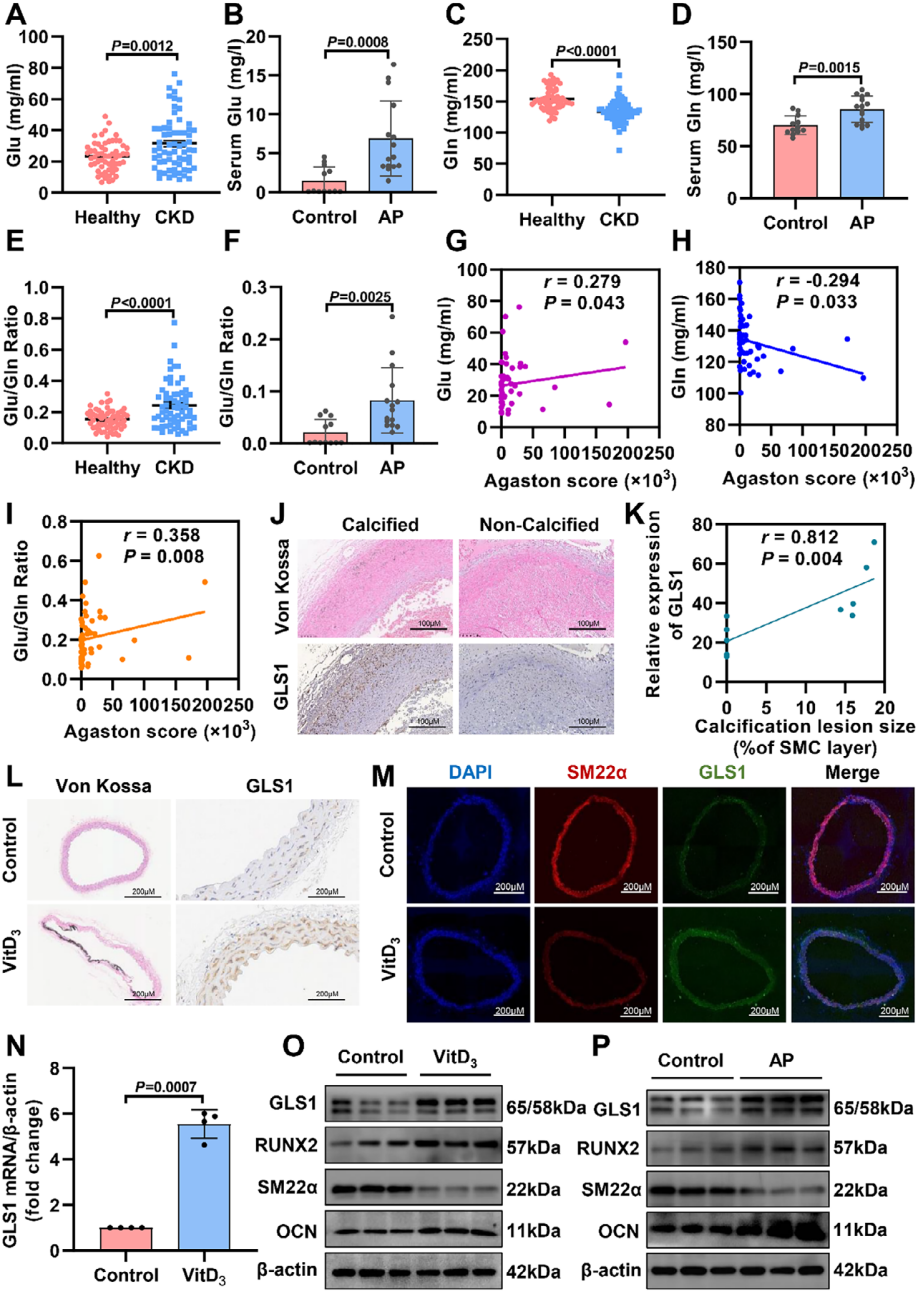
GLS1 (glutaminase 1) and its catalyzed production of glutamate are positively associated with arterial calcification. A) Plasma levels of Glu (glutamate) in patients with CKD (CKD, *n =* 65) and control donors (healthy, *n =* 58). B) Serum levels of Glu were determined in mice with either control (*n =* 12) or AP (high adenine and high phosphorus diet) (*n =* 15). C) Plasma levels of Gln (glutamine) in patients with CKD (CKD, *n =* 65) and control donors (healthy, *n =* 58). D) Serum levels of Gln were detected in control (*n =* 12) or AP (*n =* 15) mice. E) Plasma levels of Glu/Gln ratios in patients with CKD (CKD, *n =* 65) and control donors (healthy, *n =* 58). F) Analyzing serum levels of Glu/Gln ratio in control (*n =* 12) or AP (*n =* 15) mice. G–I) The correlation analysis between plasma levels of Glu, Gln, and Glu/Gln ratios and whole aortic Agatston scores in CKD patients (*n =* 53). J) Representative Von Kossa staining (upper) and immunohistochemical staining of GLS1 (lower) in arteries with or without calcification from patients (*n =* 5). Scale bar: 100 µm. K) Spearman's correlation analysis for relative expression of GLS1 and calcification lesion size (% of SMC layer) in patients with or without calcification (*n =* 5). L) Representative immunohistochemical images of GLS1 staining in control or VitD_3_ (500 IU kg^−1^, once a day for three consecutive days)‐induced arterial calcification (*n =* 3). Scale bar: 200µm. M) Representative immunofluorescence images in mice aortas from control or VitD_3_‐induced arterial calcification groups (*n =* 4). Scale bar: 200 µm. N) mRNA levels of GLS1 in aortas from control or VitD_3_‐induced arterial calcification (*n =* 4). O) The expression of GLS1 protein was confirmed from mice aortas in either the control or VitD_3_‐induced arterial calcification groups via representative immunoblots (*n =* 4). P) Representative immunoblots of GLS1 levels from mice aortas in control or AP‐induced arterial calcification groups (*n =* 5). Data are shown as means ± SEM. Two‐tailed, unpaired Student's *t*‐test was used in (A) – (F) and (N); Spearman's rank correlation coefficient was used in (G), (H), (I), (K).

KEGG pathway enrichment analysis using the MetaboAnalyst database revealed that blood metabolites causally associated with arterial calcification are primarily involved in glutamate metabolism (Figure S1A, Supporting Information). Clinical research found that CKD patients were prone to developing stroke,^[^ ^23^ ^]^ cognitive impairment,^[^ ^24^ ^]^ Alzheimer's disease,^[^ ^25^ ^]^ schizophrenia,^[^ ^26^ ^]^ and so on. Significantly, the comorbidities of the above‐mentioned diseases are all due to abnormal glutamate signaling. Arterial calcification, as the most common pathological process in CKD patients, was positively correlated with the occurrence of stroke.^[^ ^27^ ^]^ In our clinical study, the baseline characteristics of enrolled populations are shown in Table S1 (Supporting Information). Consistently, Self‐Rating Depression Scale (SDS) Score elevated with the increasing plasma glutamate (Glu) levels from CKD patients (Figure S2A, Supporting Information), and there was also a positive correlation between SDS score and arterial calcification (Figure S2B, Supporting Information). Consistently, Glu levels were upregulated in both CKD patients and AP mice (a CKD mouse model with high adenine and high phosphorus diet) (**Figure** **1**A,B). GLS1‐mediated conversion of glutamine to glutamate is a primary pathway for glutamate production. Accordingly, glutamine (Gln) levels were also measured (Figure 1C,D). We found that the ratio of Glu/Gln, regarded as an index of GLS1 activity, was dramatically increased in both CKD patients and AP mice (Figure 1E,F). Further analysis exhibited that plasma Glu levels (Figure 1G) were positively associated with calcium Agaston score (evaluated by Siemens Syngo CT Workplace software), and plasma Gln levels were inversely correlated with calcium Agaston score (Figure 1H). More meaningfully, there was a positive correlation between plasma Glu/Gln ratios and calcium Agaston score (Figure 1I). Immunohistochemical staining data exhibited that GLS1 expression from patients was enhanced in calcified arteries than in noncalcified arteries (Figure 1J). Correlation analysis indicated that the positive area of GLS1 expression was directly proportional to the positive area stained with Von Kossa (Figure 1K; Figure S3A, Supporting Information). Additionally, we evaluated the expression of GLS1 in calcified arteries from mice administered with AP diet or subjected to Vitamin‐D_3_ (VitD_3_) injection.^[^ ^28a,b,c^ ^]^ Immunofluorescence staining and immunohistochemical staining from mice also showed the increased expression of GLS1 in calcified aortic (Figure 1L,M). GLS1 mRNA was obviously elevated in calcified arteries of mice (Figure 1N). The GLS1 protein levels were strikingly enhanced in calcified aortas, as evidenced by increased osteogenic markers including RUNX2 and OCN, along with decreased contractile property marker smooth muscle‐22α (SM22α) (Figure 1O,P; Figure S3B,C, Supporting Information). Taken together, these findings implied that an upregulation of GLS1 was regarded as an essential clue to the development of arterial calcification.

### 2.2 Inhibition of GLS1 Retards the Development of Arterial Calcification In Vivo

**Figure 2:**
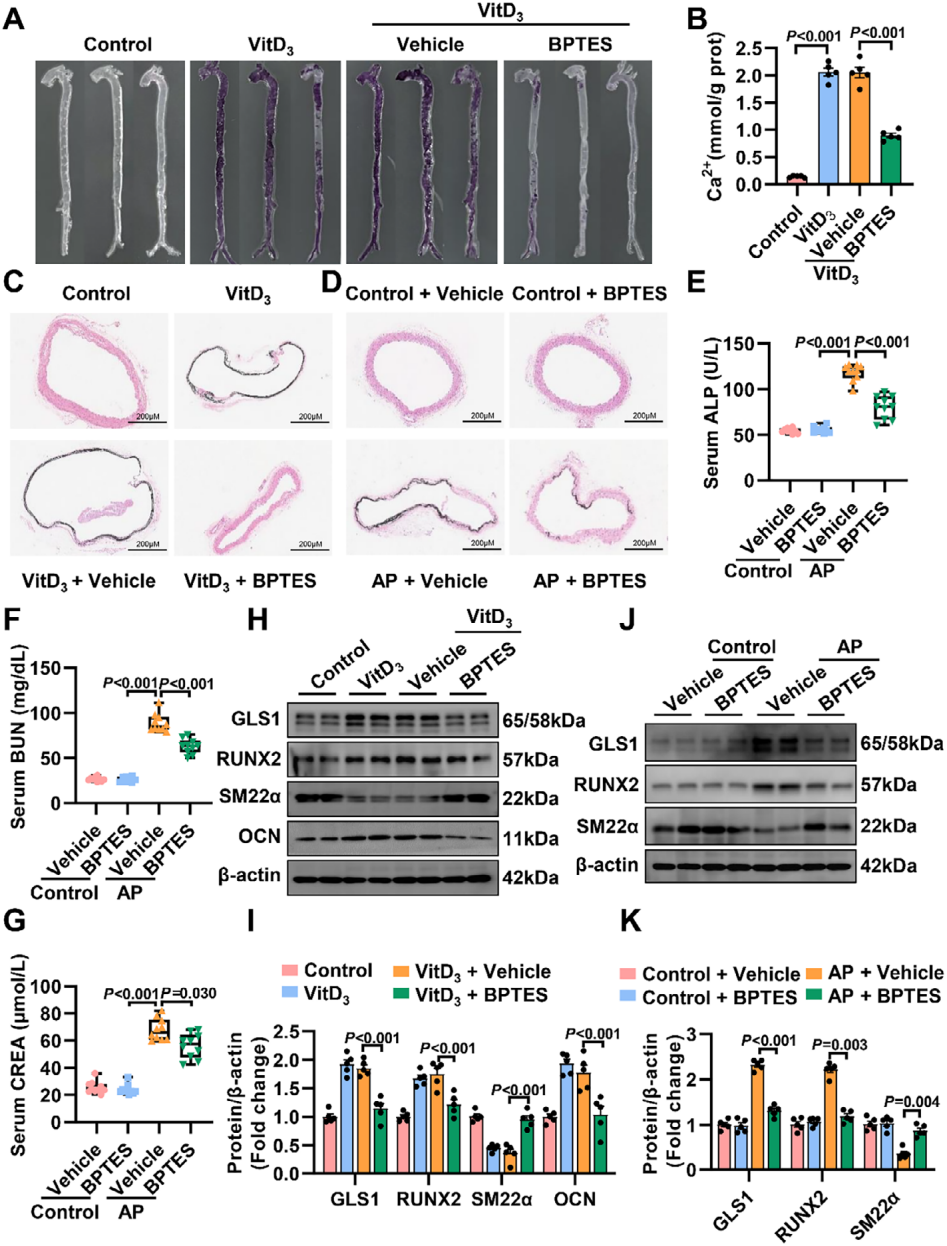
Inhibition of GLS1 retards the development of arterial calcification in vivo. A) Representative images of Alizarin Red staining from whole aortas in mice with VitD_3_ (500 IU kg^−1^)‐induced arterial calcification (*n =* 4). B) The calcium content of mice's aortas after injection with VitD_3_ is analyzed quantitatively (*n =* 5). C,D) Representative images of Von Kossa staining from the aortic ring in mice (*n =* 3). Scale bar: 200 µm. E–G) Measurement of serum ALP, BUN, and CREA levels were performed on the indicated groups of mice (*n =* 10). H,I) Representative immunoblotting and statistical diagram demonstrated that BPTES (GLS1 inhibitor, 0.25 mg/20 g/200 µL, three times a week) significantly retarded VitD_3_‐induced arterial calcification (*n =* 5). J,K) Immunoblotting analysis and statistical diagram showed that BPTES evidently attenuated CKD‐related arterial calcification (*n =* 5). Data are presented as means ± SEM. One‐way analysis of variance (ANOVA) with Bonferroni's test was used in (B), (E–G), and (K); or Dunnett's test was used in (I).

The expression of GLS1 was upregulated during arterial calcification, so it is interesting to determine whether GLS1 downregulation might alleviate the development of arterial calcification. A specific GLS1 inhibitor (BPTES, 0.25 mg/20 g/200 µL, three times a week) was used to confirm the role of GLS1 in the progression of arterial calcification in vivo. Compared to negative controls, interference with GLS1 obviously retarded arterial calcification, as determined by Alizarin Red staining (**Figure** **2**A). Calcium quantitative assay (Figure 2B) exhibited that BPTES reduced the calcium content of calcified arteries compared with VitD_3_‐induced arterial calcification. Consistently, Von Kossa staining (Figure 2C,D) also revealed that BPTES administration suppressed arterial calcification when compared to the VitD_3_ or AP group. At the same time, serum alkaline phosphatase (ALP), blood urea nitrogen (BUN) and serum creatinine (CREA), factors known to be associated with increased occurrence of arterial calcification, were also improved in mice (Figure 2E–G). In western blot analysis, osteogenic markers (RUNX2, OCN) were substantially prompted during arterial calcification, while were alleviated after GLS1 inhibition (Figure 2H–K). Taken overall, these data indicated that GLS1 deficiency retarded arterial calcification.

### 2.3 GLS1 Accelerates Osteogenic Reprogramming of VSMCs

**Figure 3:**
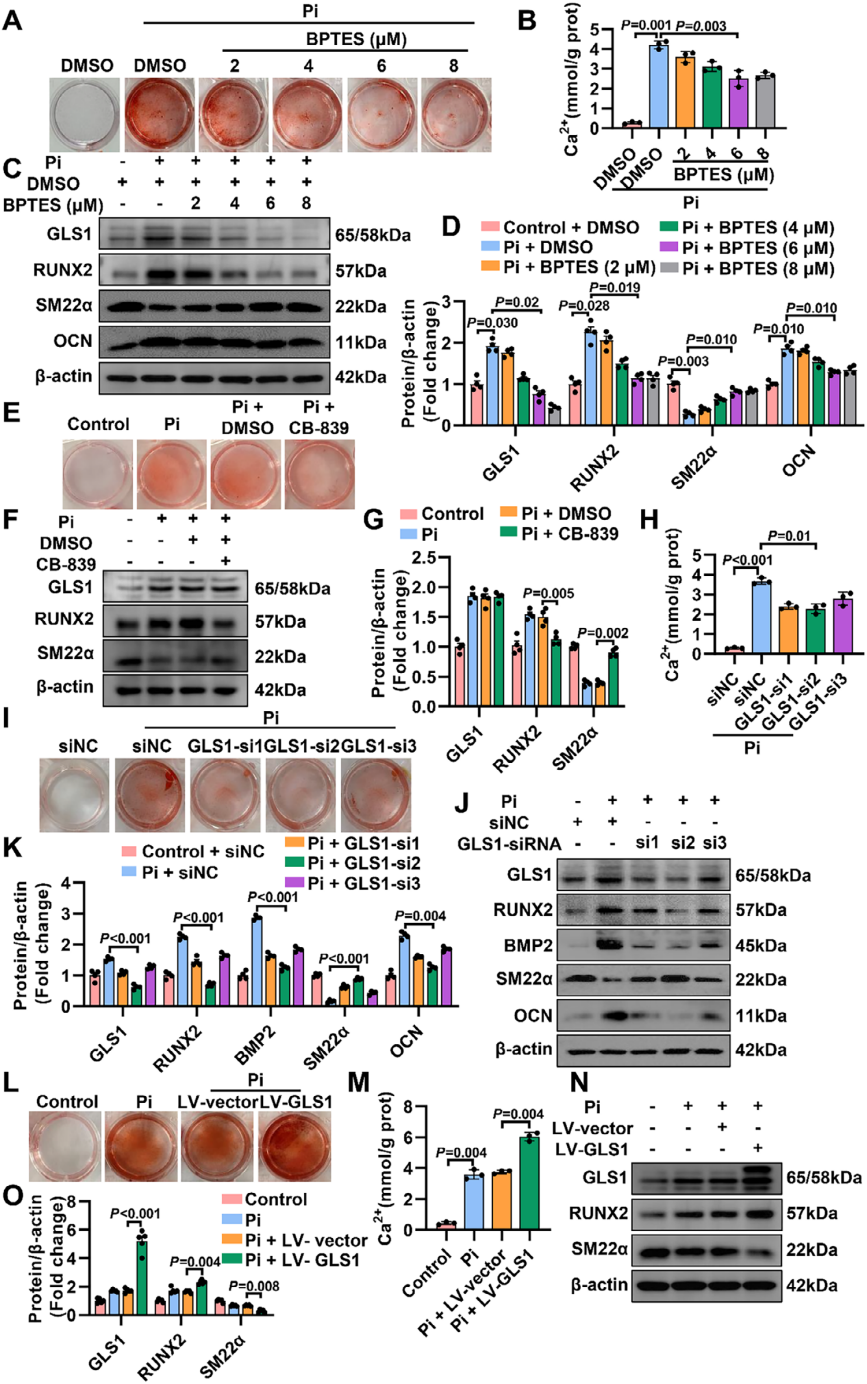
GLS1 accelerates osteogenic reprogramming of VSMCs. A) Representative images of Alizarin Red staining from VSMCs incubated with sodium phosphate dibasic (Pi, 3.0 mm) and GLS1 inhibitor (BPTES) for 7 days (*n =* 3). B) Quantitative analysis of calcium content in Pi‐mediated osteogenic transdifferentiation of VSMCs cocultured with GLS1 inhibitor (BPTES) (*n =* 3). C, D) Representative immunoblots showed the effects of GLS1 inhibitor (BPTES) on VSMCs osteogenic transdifferentiation induced by Pi (*n =* 4). E) VSMCs treated with CB‐839 (1 µm) were stained for mineralization by Alizarin Red staining (*n =* 4). F, G) VSMCs suffered osteogenic transdifferentiation caused by Pi in the absence or presence of CB‐839, as shown in a representative western blot analysis and statistical diagram (*n =* 4). H) VSMCs were pretransfected with si‐negative control (siNC) or GLS1‐siRNA (si1, si2, si3), and then exposed to Pi for 7 days before the quantitative analysis of calcium content was conducted (*n =* 3). I) Pi‐mediated osteogenic transdifferentiation of VSMCs pretransfected with siNC or GLS1‐siRNA were observed through Alizarin Red staining (*n =* 3). J, K) Pretransfected with siNC or GLS1‐siRNA, VSMCs were treated with Pi for 7 days, as depicted in a representative western analysis (*n =* 4). L) Representative images of Alizarin Red staining in Pi triggered osteogenic transdifferentiation of VSMCs preinfected with Lentivirus‐vector (LV‐vector) or Lentivirus‐GLS1 (LV‐GLS1) (*n =* 3). M) Quantitative analysis of calcium content in Pi led to osteogenic transdifferentiation of VSMCs preinfected with LV‐vector or LV‐GLS1 (*n =* 3). N, O) Representative immunoblots reflected the effects of GLS1 overexpression on VSMCs osteogenic transdifferentiation with a treatment of Pi for 7 days (*n =* 5). Data are expressed as means ± SEM. One‐way ANOVA with Bonferroni's test was used in (B), (D), (G), (H), and (M); or Dunnett's test was used in (K) and (O).

Osteogenic reprogramming of VSMCs is the key process during arterial calcification,^[^ ^29^ ^]^ so we further tested the role of GLS1 in osteogenic reprogramming of VSMCs in vitro. BPTES is an allosteric inhibitor of GLS1 that alters protein structure and may expose sites susceptible to degradation, resulting in changes in GLS1 protein levels.^[^ ^13^, ^30^ ^]^ Osteogenic reprogramming of VSMCs induced by sodium phosphate dibasic (Pi, 3.0 mm) was largely antagonized with BPTES treatment when compared with the negative control (Pi + DMSO) group by Alizarin Red staining (**Figure** **3**A; Figure S4A, Supporting Information) and quantitative analysis of calcium content (Figure 3B). Western blot results showed that the osteogenic markers were induced by Pi but were reversed after GLS1 inhibitor treatment (Figure 3C,D). CB‐839 behaved as a primarily noncompetitive inhibitor by inhibiting enzyme activity without affecting protein levels. It also exhibited the protective effect on VSMCs osteogenic reprogramming compared to the group of Pi+DMSO using Alizarin Red staining (Figure 3E; Figure S4B, Supporting Information) and western blot (Figure 3F,G). Consistently, upon Pi stimulation, GLS1‐siRNA intervention (Figure S4C,D, Supporting Information) also achieved the same anti‐calcification effect on VSMCs compared to siNC, as evidenced by calcium content assay (Figure 3H), Alizarin Red staining (Figure 3I; Figure S4E, Supporting Information) and western blot analysis (Figure 3J,K). In contrast, as a result of GLS1 overexpression (Figure S4F,G, Supporting Information), vascular mineralization was dramatically exacerbated (Figure 3L–O; Figure S4H, Supporting Information). Collectively, these data represented that GLS1 elevation acted as a promoting role in the osteogenic reprogramming of VSMCs during arterial calcification.

### 2.4 GLS1‐Related Glutamate Accelerates Osteogenic Reprogramming

**Figure 4:**
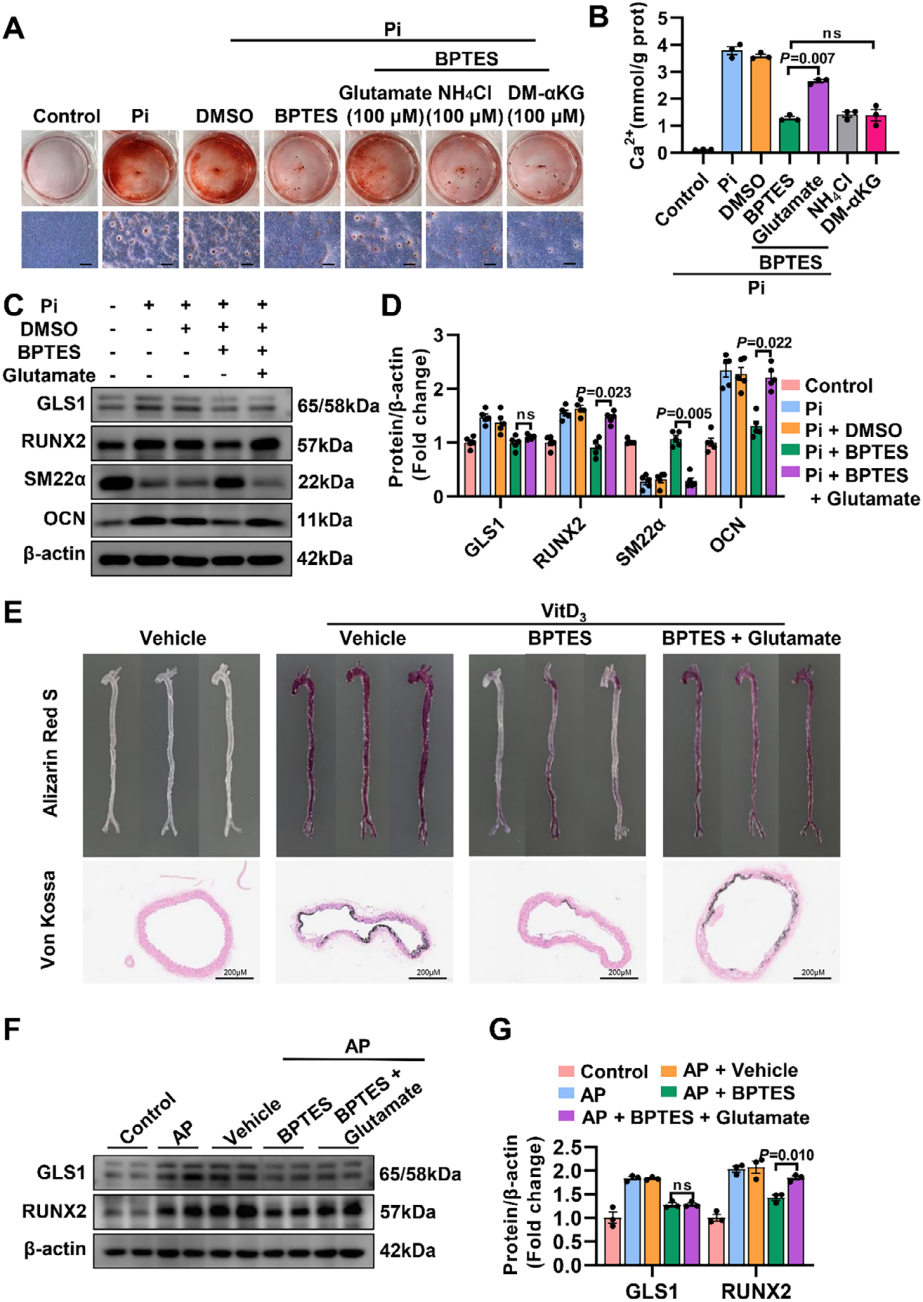
GLS1‐related glutamate accelerates osteogenic reprogramming. A) Representative images of Alizarin Red staining with replenishment of GLS1‐mediated primary metabolites on the basis of treating with GLS1 inhibitor (*n =* 3), (NH_4_Cl: a supplement of ammonia, DM‐ αKG: a supplement of αKG). Scale bar: 200 µm. B) Quantitative analysis of calcium content with replenishment of GLS1‐catalyzed primary metabolites based on treating with GLS1 inhibitor (*n =* 3). C,D) Representative immunoblots showed the effects of glutamate supplements on VSMCs osteogenic transdifferentiation in the presence of BPTES (*n =* 5). E) Representative images of Alizarin Red staining from whole aortas (upper) and Von Kossa staining from aortic ring (lower) in mice that received VitD_3_ injection (*n =* 4). Scale bar: 200 µm. F‐G) Representative immunoblotting showed the impact of glutamate replenishment on arterial calcification on the basis of exposing to BPTES in the aortas (*n =* 3). Data are shown as means ± SEM. One‐way ANOVA with Dunnett's test was used in (B), (D) and (G).

Considering that GLS1 is defined as a rate‐limiting enzyme for glutamine metabolism, the above results reflected a strong link between GLS1‐regulated glutamine metabolism and arterial calcification. GLS1‐mediated glutamine metabolism catalyzes the production of various metabolites, mainly including glutamate, ammonia, α‐ketoglutarate (αKG), etc. Therefore, we incubated VSMCs in an osteogenic medium with a GLS1 inhibitor and supplemented the medium with several downstream GLS1‐catablized metabolites, including glutamate, NH_4_Cl (a supplement of ammonia), and DM‐αKG (a supplement of αKG). As shown in **Figure** **4**A, only glutamate reversed the improved effects of VSMCs osteogenic transdifferentiation caused by BPTES. Furthermore, calcium deposition (Figure 4B) and western blot analysis exhibited a consistent trend (Figure 4C,D). To better confirm that GLS1‐regulated glutamate accelerated osteogenic reprogramming, mice injected with VitD_3_ to induce arterial calcification were treated with 2% glutamate by gavage daily. As expected, glutamate supplements dramatically deteriorated the anti‐calcification effect of BPTES in vivo (Figure 4E). Aortas were also examined using western blot analysis to validate the promoting role of glutamate in GLS1‐mediated arterial calcification (Figure 4F,G), while glutamate did not affect the level of GLS1 protein. Altogether, these findings suggested that GLS1‐related glutamate played a crucial role in aggravating the development of arterial calcification.

### 2.5 Glutamate Aggravates Osteogenic Reprogramming of VSMCs by Activating NMDAR

**Figure 5:**
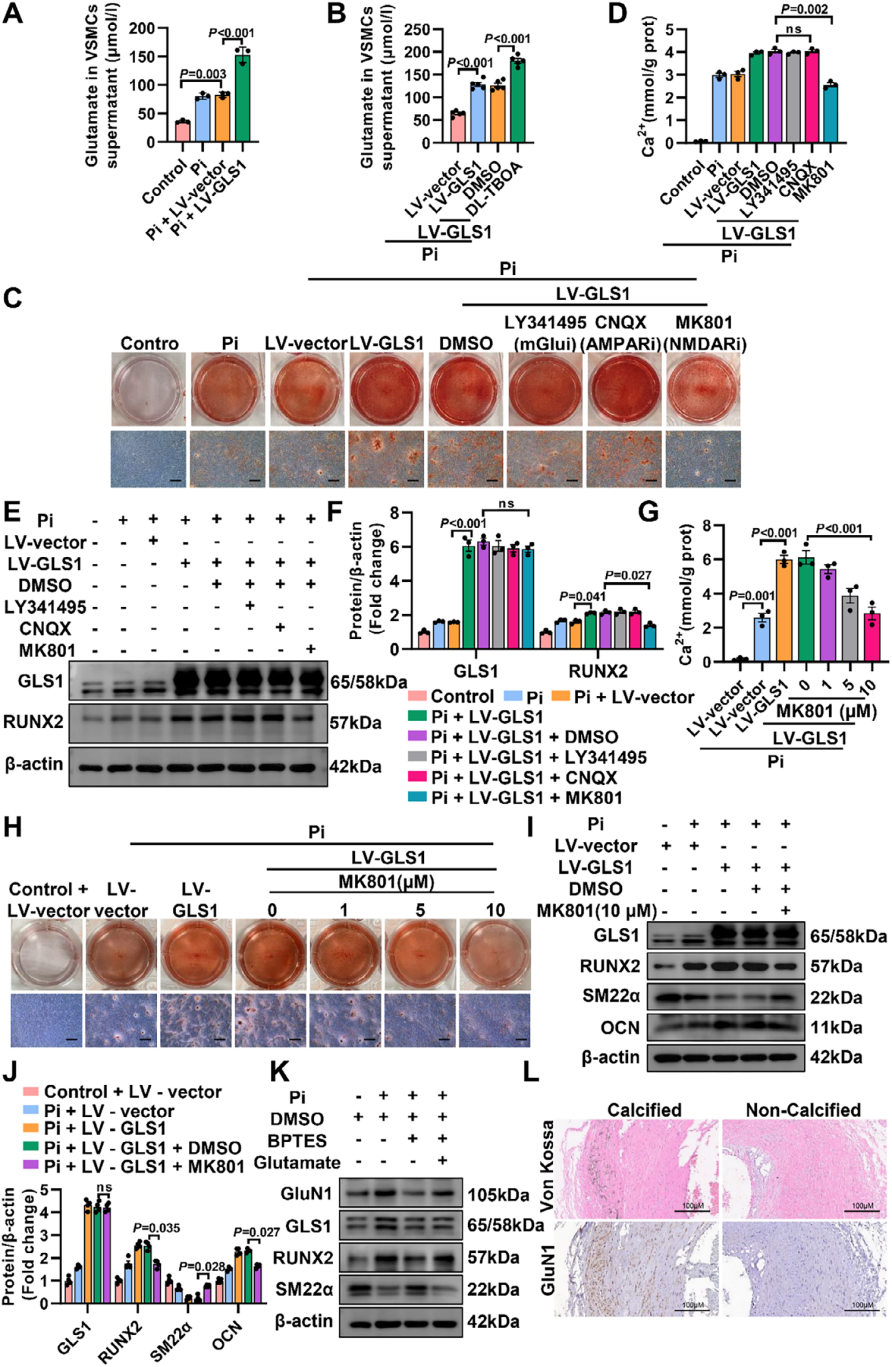
Glutamate aggravates osteogenic reprogramming of VSMCs by activating NMDAR. A) Detection of extracellular glutamate released into the supernatant of cultured VSMCs from control and Pi with or without GLS1 overexpression (*n =* 3). B) Measurement of extracellular glutamate released into the supernatant of cultured VSMCs from Pi with overexpression of GLS1 in the absence or presence of DL‐TBOA (a glutamate transport inhibitor, 100 µm) for 7 days (*n =* 5). C,D) After overexpression of GLS1, VSMCs were incubated with Pi, and then exposed to glutamate receptor inhibitors (LY341495: mGlu inhibitor, 10 µm; CNQX: AMPAR inhibitor, 10 mm; MK801: NMDAR inhibitor, 10 µm), respectively. The osteogenic reprogramming of VSMCs was investigated using Alizarin Red staining (*n =* 3) and Calcium quantitative analysis (*n =* 3). Scale bar: 200 µm. E,F) In the presence of GLS1 overexpression, western analysis of RUNX2 was performed in VSMCs treated with Pi and 3 different glutamate receptor inhibitors (*n =* 3). G,H) Dose‐response relationship for the effect of MK801 on VSMCs with overexpression of GLS1 and Pi treatment was determined by calcium content assay (*n =* 3) and Alizarin Red staining (*n =* 3). Scale bar: 200 µm. I,J) Western Blots analysis of VSMCs overexpressing GLS1 and treated with Pi, both in the absence and presence of MK801 (*n =* 4). K) The expression of GluN1 protein levels from VSMCs in the indicated group was measured by western blot assay (*n =* 5). L) Representative Von Kossa staining (upper) and immunohistochemical staining of GluN1 (lower) in arteries with or without calcification from patients (*n =* 5). Scale bar: 100 µm. Data are represented as means ± SEM. One‐way ANOVA with Bonferroni's test was used in (A), (B), (D), and (J), or Dunnett's test was used in (F) and (G).

In regard to GLS1‐related glutamate‐promoted osteogenic reprogramming of VSMCs during arterial calcification, we explored the underlying mechanism by which glutamate worked. The binding of glutamate released into the extracellular environment to its corresponding receptors on the cell membrane is a classic mode of action for glutamate.^[^ ^31^ ^]^ Therefore, we first detected the level of extracellular glutamate during osteogenic reprogramming of VSMCs. As shown in **Figure** **5**A, compared to the control group, an increase in extracellular glutamate levels is induced in VSMCs treated by Pi. More so, extracellular glutamate was further upregulated owing to overexpression of GLS1 compared with Pi treatment group. Subsequently, a glutamate transporter inhibitor (DL‐TBOA) was employed, and the operation brought about a noteworthy accumulation in glutamate levels in VSMCs supernatant (Figure 5B). Consistently, overexpression of GLS1 exacerbated the calcification of VSMCs, and simultaneous treatment with DL‐TBOA further enhanced this pro‐calcification effect by GLS1, as evidenced by Alizarin Red staining (Figure S5A, Supporting Information). All these results suggested that there are functional glutamate receptors for sensing extracellular glutamate and facilitating downstream signaling transduction to promote calcification. Traditionally, glutamate binds and activates to its receptor to exert functions.^[^ ^31^ ^]^ Several antagonists targeting glutamate receptors were added to the osteogenic medium. Interestingly, only the NMDAR antagonist (MK801) reversed the accelerated function of GLS1‐overexpression‐induced VSMCs osteogenic reprogramming (Figure 5C–F), and the further experiments exhibited the anti‐calcification effect of MK801 in a dose‐dependent manner (Figure 5G–J). Notably, MK801 treatment did not affect the level of GLS1 protein. As a multimeric, the subunit of GluN1 determined the activity and availability of total NMDAR. As expected, mRNA (Figure S5B, Supporting Information) and protein of GluN1 (Figure 5K; Figure S5C, Supporting Information) in trend were consistent with those of the osteogenic marker (RUNX2). Besides, GluN1 expression was significantly elevated in calcified arteries from patients (Figure 5L; Figure S5D,E, Supporting Information). This represented the first indication that the expression and activity of NMDAR, especially GluN1, were upregulated in arterial calcification, playing a worsening role in its progression. In a word, activation of NMDAR by GLS1‐related glutamate was indispensable for GLS1‐caused VSMCs osteogenic reprogramming.

### 2.6 Glutamate‐Activated NMDAR Induces Nuclear Translocation of *β*‐Catenin by Mediating Ca^2+^ influx

**Figure 6:**
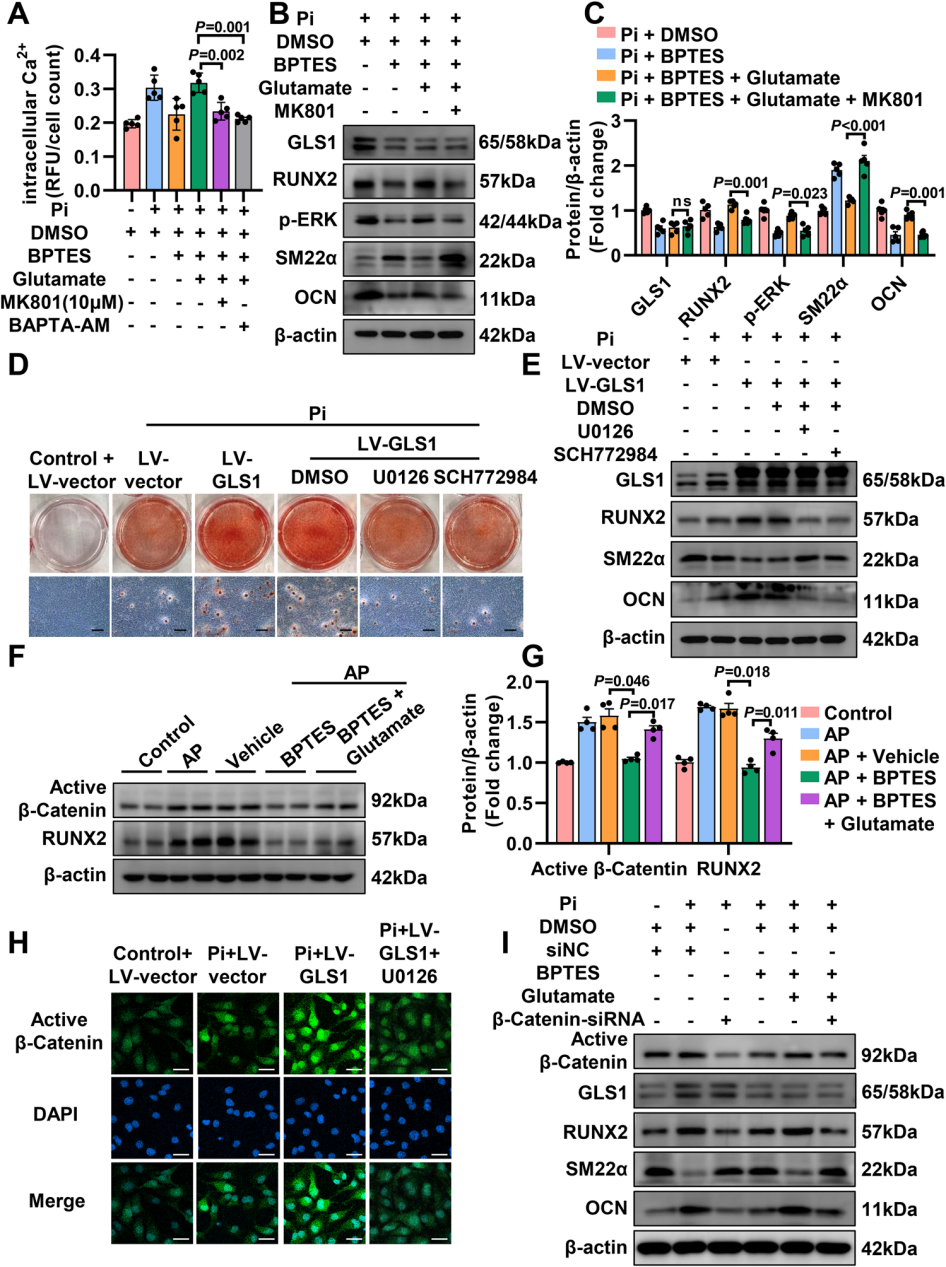
Glutamate‐activated NMDAR induces nuclear translocation of *β*‐Catenin by mediating Ca^2+^ influx. A) Measurement of intracellular Ca^2+^ concentration in VASMCs after the indicated treatment (*n =* 5). B,C) Representative immunoblotting and statistical diagram depicted the levels of p‐ERK proteins following the indicated treatment (*n =* 5). D) Alizarin Red staining was conducted to observe the osteogenic reprogramming of VSMCs treated with Pi and overexpressing GLS1 in the absence or presence of ERK inhibitors (U0126, 10 µm; SCH772984, 10 µm) in vitro (*n =* 5). Scale bar: 200 µm. E) Representative western blotting showed the effects of ERK inhibitors (U0126 and SCH772984) on osteogenic reprogramming of VSMCs treated with Pi and overexpressing GLS1 (*n =* 5). F,G) Western Blot analysis of active *β*‐Catenin protein expression from aortas in mice following the indicated treatment (*n =* 4). H) Changes in nuclear translocation of active *β*‐Catenin were shown by representative immunofluorescence images (*n =* 4). Scale bar: 20 µm. I) Representative immunoblotting of active *β*‐Catenin protein levels with indicated treatment (*n =* 4). All values are expressed as means ± SEM. One‐way ANOVA with Bonferroni's test was used in (A), and Dunnett's test was used in (C) and (G).

Activation of NMDAR facilitates calcium influx.^[^ ^32^ ^]^ As predicted, BPTES did lead to a reduction in intracellular calcium (**Figure** **6**A). Further to that, glutamate supplements prevented BPTES‐antagonized calcium influx during osteogenic reprogramming of VSMCs, and MK801 completely abolished the aforementioned effect. Moreover, BAPTA‐AM (an intracellular calcium chelator) intervention reversed the intracellular Ca^2+^ induced by the activation of the glutamate‐NMDAR axis. Ca^2+^ acts as a secondary messenger to activate a series of kinases.^[^ ^33^ ^]^ Thus, the fluctuations of kinase were detected. Western blot analysis showed that, compared to Pi treatment alone, phosphate‐ERK (p‐ERK) protein levels in VSMCs were down‐regulated by BPTES treatment. Furthermore, on the basis of BPTES treatment, these levels were re‐stimulated by the addition of glutamate and further inhibited by MK801 (Figure 6B,C). Surprisingly, changes of p‐ERK levels across different treatments were the most significant among the series of kinase detected (Figure S6A,B, Supporting Information). Notably, ERK inhibitors (U0126 and SCH772984) also attenuated the effect of osteogenic reprogramming of VSMCs with GLS1 overexpression (Figure 6D,E; Figure S6C,D, Supporting Information). It concluded that GLS1‐related glutamate promoted osteogenic reprogramming of VSMCs by activating NMDAR mediated‐Ca^2+^ influx, and further facilitated activation of ERK. It is well established that activated ERK is associated with the stability of *β*‐Catenin, thereby promoting its nuclear translocation.^[^ ^34a,b^ ^]^ Stimulated by various factors, *β*‐catenin on the cell membrane is released to the cytoplasm, where its active form (nonphosphorylated *β*‐Catenin) is stabilized and translocated to the nucleus, functioning as a transcriptional activator of downstream genes such as RUNX2 and MSX2.^[^ ^20^, ^21^, ^22^, ^35^ ^]^ Accordingly, we investigated the change of active *β*‐Catenin protein. It showed that BPTES administration markedly reduced the level of active *β*‐Catenin protein, whereas additional glutamate supplement promoted the increased expression of its active form (Figure 6F,G). Furthermore, we conducted the nuclear/cytoplasmic fractionation experiment to examine the subcellular distribution of *β*‐Catenin and observed the increased nuclear active *β*‐Catenin in VSMCs treated with Pi and incubated with BPTES as well as glutamate, compared to Pi and BPTES treatment only (Figure S7A,B, Supporting Information). Similarly, after the glutamate supplement, increased active *β*‐Catenin protein was also observed in VSMCs cocultured with Pi and CB‐839 (Figure S7C,D, Supporting Information). Then, MK801 was employed to test whether *β*‐Catenin was regulated by the related glutamate‐NMDAR axis. As predicted, MK801 was resistant to the upregulation of active *β*‐Catenin protein (Figure S8A,B, Supporting Information). Concurrently, ERK inhibitors displayed the same trend as MK801 (Figure S9A,B, Supporting Information). As shown in immunofluorescence images (Figure 6H), a low level of active *β*‐Catenin was observed in untreated cells. Under Pi treatment, the level of active *β*‐Catenin was upregulated in both cytoplasm and nucleus, especially in the nucleus. With the overexpression of GLS1, active *β*‐Catenin levels in nucleus were further increased compared to Pi group, indicating the increased nuclear translocation promoted by GLS1. In contrast, ERK inhibitor counteracted the promoting effect of GLS1 on active *β*‐Catenin levels in the presence of Pi treatment. In addition, the effect of overexpression GLS1 in VSMCs was alleviated by silencing *β*‐Catenin (Figure 6I; Figure S10A–C, Supporting Information). Accordingly, GLS1 triggers osteogenic reprogramming of VSMCs in a *β*‐Catenin manner.

## 3 Discussion

**Figure 7:**
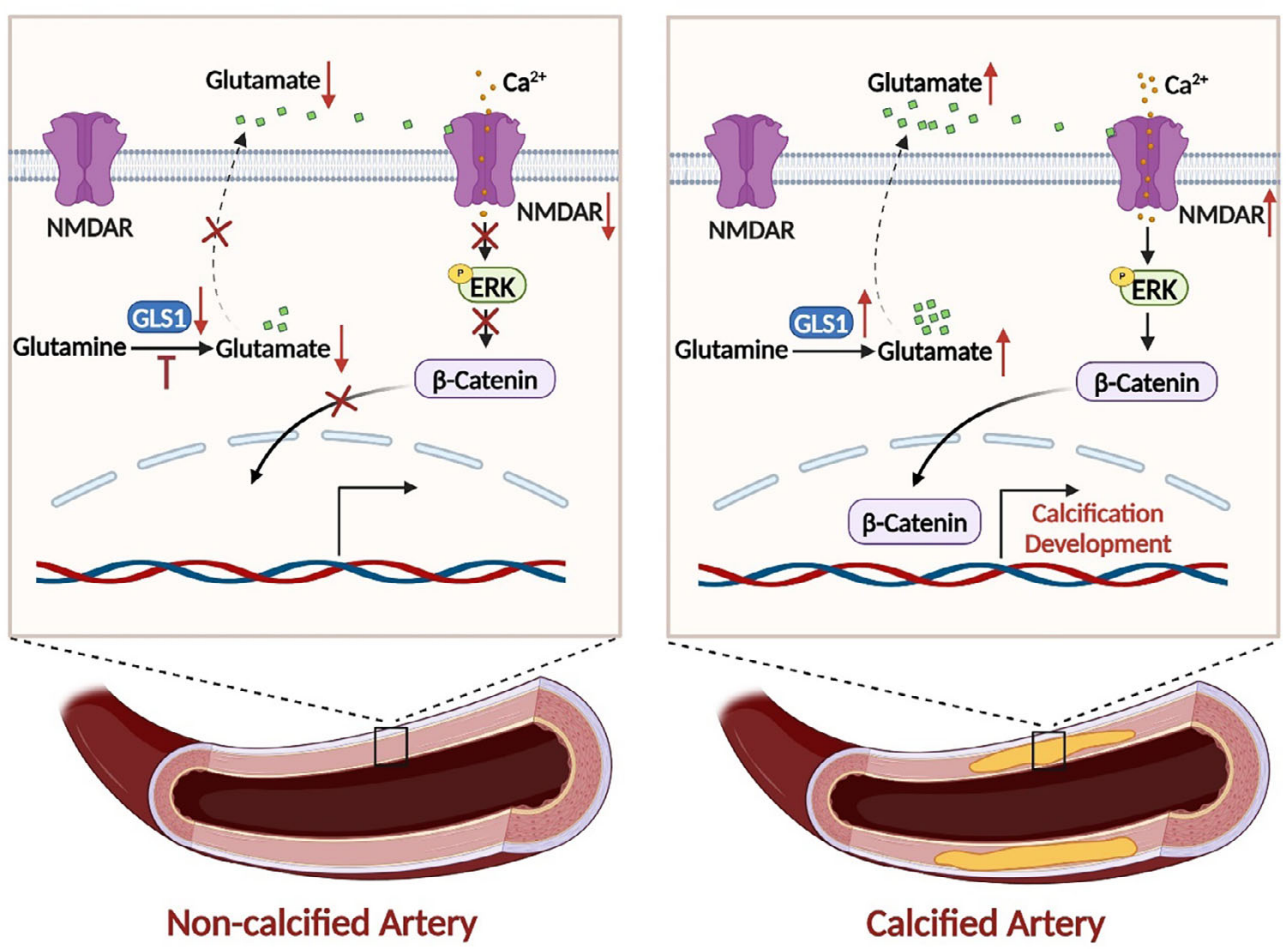
Schematic illustration of GLS1‐catalyzed glutamate production in promoting arterial calcification via activating NMDAR/Ca^2+^/*β*‐Catenin pathway. GLS1 as a novel contributor to arterial calcification. GLS1‐catalyzed glutamate exerts the promoting effects on osteogenic reprogramming in arteries. NMDAR, a glutamate receptor, is also activated and overexpressed in arterial mineralization. GLS1 redundancy‐mediated glutamate superfluity promotes calcium influx and subsequent ERK phosphorylation by activating NMDAR, leading to nuclear translocation of *β*‐Catenin and thus progression of arterial calcification.

In this present study, we demonstrated a previously unidentified role of GLS1 in the development of arterial calcification. Above all, the increased plasma ratio of Glu/Gln, a reflection of GLS1 activity, was first recognized in CKD patients compared with healthy controls. More so, plasma levels of Glu and Glu/Gln ratio were positively correlated with the progression of arterial calcification. Further experiments represented that inhibition or knockdown of GLS1 alleviated VSMCs osteogenic reprogramming and the subsequent arterial calcification, which were reversed by glutamate supplementation in vivo and in vitro. Mechanistically, GLS1‐mediated glutamate activated NMDAR, an unidentified signaling pathway in the field of arterial calcification, to promote Ca^2+^ influx and consequent ERK activation, leading to arterial calcification by promoting nuclear translocation of *β*‐Catenin. A schematic in **Figure** **7** illustrates GLS1‐catalyzed glutamate production in promoting arterial calcification.

GLS1, the first rate‐controlling enzyme in the glutaminolysis pathway, converts glutamine to glutamate to yield multiple metabolites for anabolism in various cells.^[^ ^36^ ^]^ GLS1‐regulated glutamine metabolism has been shown to nourish various cardiovascular diseases. GLS1 inhibitor (CB‐839) was effective in reducing vascular collagen content, vascular stiffness, and proliferation, as well as histologic and hemodynamic parameters of pulmonary hypertension.^[^ ^37^ ^]^ Furthermore, BPTES partially prevented aortic valve thickness, fibrosis, and calcification.^[^ ^13^ ^]^ Inhibition of glutaminolysis (CB‐839) in activated myofibroblasts, protects against cardiac functional decline and fibrosis.^[^ ^12^ ^]^ GLS1‐related glutaminolysis aggravates age‐associated disorder. BPTES administration ameliorated age‐associated glomerulosclerosis, kidney dysfunction, lung fibrosis, SA–*β*‐gal–positive population, and improved age‐associated weakness of grip strength and short‐hanging endurance in aged mice.^[^ ^9^ ^]^ In addition, glutaminolysis‐related productions were reported to promote osteogenic differentiation of mesenchymal stem cells.^[^ ^14^, ^38^ ^]^ Given the above situation, whether glutaminolysis is involved in the pathogenesis of arterial calcification attracts our attention. Therefore, assessed by the level of plasma Glu/Gln ratio, the GLS1 activity was higher in CKD patients than in healthy controls for the first time. Furthermore, the plasma Glu/Gln ratio was positively associated with aortic Agaston calcium score in CKD patients. It was consistent with a universal upregulation in GLS1 expression in the arteries from calcified patients or calcified mice. To further clarify the role of GLS1 in arterial calcification, GLS1 inhibitor administration has been proven to significantly impede arterial calcification. These results indicate the effect of VSMCs GLS1 abundance on calcific vasculopathy. It is suggested that targeting GLS1 serves as a new insight into antagonistic arterial calcification.

GLS1‐regulated glutaminolysis derives an abundance of metabolic products, the primary metabolite such as glutamate, ammonium, and αKG.^[^ ^39^ ^]^ Recent studies have reported that ammonium derived from GLS1‐driven glutaminolysis was responsible for age‐associated disorder. Enhanced GLS1‐mediated glutaminolysis induced ammonia production, which neutralized the lower pH and improved the survival of the senescent cells.^[^ ^9^ ^]^ Additional research illustrated that glutaminolysis‐related glutamate derivatives including αKG, proline, and asparagine promoted the proliferation of MSCs and osteogenic differentiation.^[^ ^14a,b^ ^]^ A study has revealed that GLS1‐mediated glutamate was associated with the inflammation in the development of psoriasis.^[^ ^40^ ^]^ In our research, we conducted incubated experiments using the original and primary metabolites to conclude that only GLS1‐induced glutamate was the culprit of VSMC osteogenic reprogramming. Conversely, overexpression of GLS1‐induced glutamate sufficiency pushed VSMCs osteogenic reprogramming and consequence arterial calcification. Moreover, glutamate administration in vivo also showed that glutamate was the initiator of glutaminolysis‐facilitated arterial calcification, which was consistent with previous research.^[^ ^18^ ^]^ The reason why different metabolites catalyzed by GLS1 play different roles may be due to different cell types and stimulation conditions. It is suggested that under the state of GLS1‐mediated glutamine metabolism, the same amount of glutamate is more harmful than ammonia, which has drawn our attention. Further experiments are needed to verify the deeper work. Given the above evidences, glutamate as a major component of a ginomoto, may shed light on intake of ginomoto in daily diet.

Glutamate is a ligand, which is decoded by an array of glutamate receptors to realize diverse functions. To investigate the receptor of responsibility without bias, multiple inhibitors for glutamate receptors were employed. Interestingly, only treatment with MK801, an NMDAR inhibitor, showed the anti‐calcification effect despite increased glutamate mediated by GLS1 overexpression, indicating that the glutamate‐NMDAR axis was responsible for GLS1‐related glutamate evoking arterial calcification. Hinoi E et al. found that sustained exposure to the NMDAR antagonist (MK801) significantly impeded osteogenesis in osteoblast.^[^ ^18^ ^]^ NMDAR is assembled as tetramers, and modulation of GluN1 plays a key role in controlling the total NMDAR pool available for activation.^[^ ^41^ ^]^ Dumas et al. revealed that NMDAR exists in human pulmonary arterial smooth muscle cells, of which GluN1 overexpressed and overactivated to stimulate the development of PAH.^[^ ^42^ ^]^ Consistent with this, we also detected an increase in GluN1 protein during arterial calcification. In conclusion, these results displayed a decisive role of the glutamate‐NMDAR axis in GLS1‐mediated arterial calcification.

NMDAR activation evokes Ca^2+^ influx,^[^ ^31^, ^32^, ^41^ ^]^ and we also found the increased Ca^2+^ influx with overexpression of GLS1. Thus, several Ca^2+^‐dependent protein kinases were further detected. Interestingly, only activated ERK was significantly detected, while inhibiting ERK attenuated the effect of GLS1 on arterial calcification, indicating that ERK served as the bridge of GLS1‐related glutamate/NMDAR axis‐induced arterial calcification. Activated ERK promotes the stability of active *β*‐Catenin and its translocation to nucleus.^[^ ^34a^ ^]^ As presented in our immunofluorescence results, accumulation of active *β*‐Catenin was transported to nucleus. Therefore, *β*‐Catenin was also confirmed as a major role of GLS1 mediated arterial calcification. This effect may be related to the binding of TCF/LEF with *β*‐Catenin in nucleus, and the subsequent stimulation of the transcription of RUNX2.

The present study shows that GLS1 is a deteriorating regulator in arterial calcification through glutamate products. It is worth noting that the activation and upregulation of GluN1 in arterial calcification are also reported for the first time. However, the regulatory mechanism between GluN1 and arterial calcification is yet unclear. In addition, potential confounding factors of the enrolled population, such as the patient's diet and basic metabolic condition, were not controlled in this study. Further clinical trials are needed to address these confounding factors and reduce the impact on the study outcomes.

In conclusion, clinical investigation emphasized the correlation of increased plasma glutamate and GLS1 activity with the development of arterial calcification. We identified the initiating factor of GLS1 in promoting the process of arterial calcification. Further speaking, GLS1‐related glutamate, NMDAR activation that was verified as an unidentified regulatory molecule in vascular calcification, accelerated the osteogenic reprogramming of VSMCs and induced arterial calcification progression in a *β*‐Catenin manner. These findings provide novel perspectives on the prevention and inhibition of clinical arterial calcification.

## 4 Experimental Section

### Clinical Study Population and Human Artery Samples

This cross‐section clinical study was conducted from July 2023 to January 2024 at The Eighth Affiliated Hospital of Sun Yat‐sen University, Shenzhen, China. A total of 123 participants (58 healthy subjects and 65 CKD patients) were enrolled, and their plasma samples were detected by LC‐MS/MS. Among CKD patients, 12 individuals lacked chest CT data. For the remaining 53 patients with available CT data, the statistical analysis of calcification scores was performed using the calcium Agaston score. The diagnostic criterion of CKD^[^ ^43^ ^]^ was based on eGFR decline persisting for 3 months or longer, and specific manifestation of eGFR less than 60 mL min^−1^/1.73 m^2^. Patients were excluded if they had acute kidney disease (AKD), elevated liver enzymes, acute cardiovascular diseases (atrial fibrillation, myocardial infarction, unstable angina, heart failure, and stroke), malignancies, acute infectious diseases, history of taking α‐ketoglutarate or phosphate binders, vitamin D analogs, or the corticosteroids within 3 months before recruitment, were currently pregnant or wished to get pregnant throughout the study. Healthy participants were matched age and gender, and excluded if they had CKD, AKD, elevated liver enzymes, acute cardiovascular diseases (atrial fibrillation, myocardial infarction, unstable angina, heart failure, and stroke), malignancies, acute infectious diseases, history of taking α‐ketoglutarate or corticosteroids within 3 months before recruitment. Artery samples were originated from patients who underwent an operation of renal cell carcinoma. The clinical trial protocol and human samples collection were performed with the Declaration of Helsinki and approved by the Ethics Committee of The Eighth Affiliated Hospital of Sun Yat‐sen University (2023‐100‐01). Informed Consent Form have been obtained from all participants or legal representatives.

### Assessment of Calcium Score

Among the cohort, CKD patients received a chest CT scan and calculated Agatston scores to quantify the severity of aortic calcification. The CT images were reconstructed using slices of 1 mm thickness to evaluate aortic calcification with Siemens Syngo CT Workplace software. Calcification score is calculated using the formula based on the weight coefficients corresponding to CT values.^[^ ^44^ ^]^ More details have been described in previous research.^[^ ^45^ ^]^ Arterial calcification was identified as an Agatston score or Volume score greater than 0.

### LC‐MS/MS Measurement

Peripheral venous blood sample was collected from participants for at least 8 h of fasting. The operation procedure of plasma was adhered to a centrifugal speed of 3000×g for 15 min at 4 °C, followed by 5000×g for 10 min, and plasmas were accumulated for subsequent research or stored at −80 °C until analysis. LC‐MS/MS detection for samples was operated by Wuhan Metware Biotechnology Co., Ltd. (www.metware.cn). The final results were provided. The operating parameters of LC‐MS/MS summarized are listed in **Table** **1**.

**Table 1:** Operating parameters of LC‐MS/MS.

Parameter	Amide
Liquid Chromatography	LC‐ESI‐MS/MS system (UPLC, ExionLC AD, https://sciex.com.cn/)
Column	Waters ACQUITY UPLC BEH Amide (2.1 × 100 mm, i.d.1.7 µm)
Temperature	40 °C
Injection Volume	2 µL
Flow Rate	0.4 mL min^−1^
Mobile Phase A	Water with 10 mm Ammonium acetate and 0.3% Ammonium hydroxide
Mobile Phase B	90% acetonitrile/water (V/V)
Gradient	The gradient was started at 95% B (0–1.2 min), decreased to 70% B (8 min), 50% B (9–11 min), finally ramped back to 95% B (11.1–15 min)
Mass Spectrometry	AB Sciex QTRAP 6500+ System, (https://sciex.com/)
Ion Source	ESI±
Source Temperature	550 °C
Ionspray Voltage	Positive, 5500 V; Negative, −4500 V
Ion Source Gas1	45 psi
Ion Source Gas2	55 psi
Curtain Gas	35 psi
Scan Type	Scheduled multiple reaction monitoring

### Animal Experiments

All animal experiments abided by animal ethical standards and were approved by the Ethics Committee of Zhongshan School of Medicine, Sun Yat‐sen University (SYSU‐ACUC‐2022‐B0053). 8‐week‐old C57BL/6J male mice weighing 20–25 g were purchased from the Laboratory Animal Center of Sun Yat‐Sen University. Mice were housed in a temperature (23–25 °C) and humidity‐controlled colony room, maintained on a 12‐h light/dark cycle (08:00–20:00 light on). Mice were randomly divided into each group (*n =* 15). For CKD‐related‐arterial calcification, mice were exposed to a high adenine (0.15%) and high phosphorus (1.9%) diet (GDMLAC, AP diet) for 12 w. Alternatively, Vitamin D_3_ (Sangon Biotech, 500 IU kg^−1^) was intraperitoneally injected into mice continuously for 3d to induce arterial calcification. Mice were maintained on a standard chow diet until sacrifice on day 10. Meanwhile, On the first day of inducing arterial calcification, mice were intraperitoneally injected with BPTES (Selleck, 0.25 mg/20 g/200 µL) or vehicle control (200 µL of 10% DMSO in corn oil) three times a week until they were euthanized. In addition, glutamate treatment was given a daily gavage of 2% glutamate (Aladdin) dissolved in water. Animals were anesthetized with isoflurane and then euthanized by cervical dislocation under deep anesthesia.

### Assessment of Mice Serum Glutamate and Glutamine

Mice serum was centrifuged and collected for measurement of glutamate and glutamine using a Yellow Spring Instrument 2950D‐1 (YSI Inc./Xylem Inc., Yellow Springs).

### Cell Culture

Primary human VSMCs were purchased from the American Type Culture Collection (ATCC) and cultured in high‐glucose Dulbecco's modified Eagle's medium (DMEM, Gibco) containing 10% fetal bovine serum (FBS, MIKX), 1% penicillin/streptomycin (P/S, Gibco). VSMCs at passages 3–10 were handled in all cell experiments. To induce VSMCs calcification, 3.0 mm sodium phosphate dibasic (Pi, Sigma–Aldrich) was added to the cellular culture medium for 7d. The medium was changed every 2 or 3 days.

VSMCs were transfected with small interfering RNA (siRNA), GLS1 siRNA, or *β*‐ Catenin siRNA according to a direction of lipofectamine 3000 regent (Invitrogen). The sequences of siRNA are listed in Table S2 (Supporting Information). For overexpression, VSMCs were infected with lentiviral vector and lentiviral GLS1 following the manufacturer's instructions for lentivirus usage. The efficiencies of transfection or infection were identified by real‐time polymerase chain reaction (PCR) or Western blot. Treatments were carried out simultaneously with Pi stimulation: BPTES (Selleck); CB‐839 (Selleck, 1 µm); MK801 (APExBIO, 10 µm); CNQX (APExBIO, 10 µm); LY341495 (APExBIO, 10 µm); SCH772984 (APExBIO, 10 µm); U0126‐EtOH (APExBIO, 10 µm); BAPTA‐AM (Selleck, 5 mm); DL‐TBOA (MCE, 100 µm) was selected as the working concentration.

### Western Blot

Tissue or VSMCs samples were added with RIPA lysis (Beyotime) containing phosphatase (Roche PhoSTOP) and protease inhibitors (Beyotime). The final concentration of protein was analyzed using BCA Protein Assay Kits (Thermo Fisher Scientific). Proteins with different molecular weights were separated on sodium dodecyl sulfate (SDS)–polyacrylamide gel (Epizyme), and then transferred to PVDF membrane (Millipore). The PVDF membranes were blocked with 5% BSA solution at room temperature for 1 h, followed by incubation with specified antibodies overnight at 4 °C. After incubating secondary antibodies, the membranes were developed with Chemiluminescence imaging system (ChemiDoc Touch or Amersham Imager 600 or minichemi910). The bands’ intensity was analyzed by Image J software. The antibodies are listed in Table S3 (Supporting Information).

### RNA Extraction, Reverse Transcription, and Quantitative Real‐Time Polymerase Chain Reaction (qPCR)

Total RNA was extracted by RNA Purification Kit (EZBioscience) according to the manufacturer's protocols. RNA concentration was tested using Nanodrop 2000. For qPCR analysis, cDNAs were obtained and real‐time PCR amplifications were performed according to the protocol (EZBioscience). The relative expressions were represented as Ct values standardized by ACTB, and the fold changes were assessed by the 2^−ΔΔCt^ method. The sequences of primers are listed in Table S4 (Supporting Information).

### Calcification Quantification

Calcification of the whole‐mount aorta, from arcus aortae to the bifurcation of iliac artery, was determined by Alizarin Red staining (leagene). Artery tissues were fixed in 95% ethanol for 24 h, then stained with 0.003% Alizarin Red in 1% sodium hydroxide (Guangzhou) for 30 h. Two percent sodium hydroxide was used to wash tissue twice before taking photos. Calcium depositions of aorta were evaluated with the Calcium Assay Kit (Nanjing Jiancheng), which was based on the released calcium after decalcified with 0.6 mmol/l hydrochloric acid (Guangzhou) at 37 °C for 48 h. The contents of calcium were calibrated to the dry weight of artery tissues. Aortic ring slice with calcification was determined by the Von Kossa staining. Briefly, aortas were treated with 5% silver nitrate (Macklin) and exposed to ultraviolet light for 30 min, rinsed, and incubated with 5% sodium thiosulfate (GENMED) to show the staining brown. As for VSMCs calcification, was also identified by Alizarin Red staining (leagene) as previously described.^[^ ^28^, ^45^ ^]^ Calcium deposition of VSMCs is detected using a Calcium Assay Kit (Nanjing Jiancheng) after decalcifying. The contents of calcium were corrected for protein contents.^[^ ^45^ ^]^

### Detection of Glutamate in Supernatant

The concentration of glutamate in the supernatant was detected by Glutamate Assay Kit‐WST (DOJINDO). In general, 100 µL supernatant was taken from the cell culture medium, and diluted with ultrapure water. The experiment was conducted according to the manufacturer's protocol.

### Intracellular Ca^2+^ Measurement

VSMCs counts were adjusted to 3000/well in a 96‐well black opaque plate and gave the corresponding interventions. Fluo‐4‐AM (Beyotime) was added to a final concentration of 2.5 µm. The solution was gently mixed and incubated for 30 min at 37 °C in the dark. Cleaning it up and detecting it with the fluorescence microplate reader.

### Nuclear‐Cytoplasmic Separation

Nuclear and cytoplasmic separation was carried out according to the protocol (Beyotime). Briefly, cells were collected in a 1.5 mL centrifuge tube and centrifuged to obtain cell precipitation. The cells were re‐suspended in cytoplasmic lysis buffer and rested on the ice for 20 min. The supernatant was taken as cytoplasmic part after high‐speed centrifugation (4 °C, 15 000 g for 5 min). The remaining cell precipitates were suspended in the nuclear protein extraction reagent and were completely mixed at low temperature. The supernatant was taken as a nuclear component after high‐speed centrifugation (4 °C, 15 000 g for 10 min).

### Immunohistochemical Staining

Aortic tissues were fixed in 4% paraformaldehyde and embedded in paraffin to be cut into 5‐µm sections. After being baked, deparaffinization and hydration were performed with xylene and alcohol. Then, heat‐induced antigen retrieval unmasked aortic tissues. Three percent H_2_O_2_ was used to eliminate endogenous peroxidase activity. The sections were blocked in 5% BSA (Solarbio) at room temperature for 30min. After that, the regions of aorta were incubated with specified primary antibodies overnight at 4 °C and enhanced enzyme‐linked goat anti‐mouse/rabbit IgG polymer (Zhongshan Company) at room temperature for 30min. Diaminobenzidine was selected for visualization. After being hydrated again and sealed, images were captured.

### Immunofluorescence Staining

Immunofluorescence observation of GLS1 and SM22α in mouse aortic tissue was carried out. Parts of the details are described in the section of Immunohistochemical staining. After being incubated with primary antibodies, Alexa Fluor 594–conjugated secondary antibodies (Beyotime) and Alexa Fluor 488–conjugated secondary antibodies (Beyotime) were chosen to incubate. Finally, containing DAPI (Servicebio) sealing agent was stained nuclei. Microscopy was performed with Leica. As for VSMCs, 1× PBS was used to wash 3 times after treatments. Then, 4% paraformaldehyde solution was applied to fix for 15 min. Next, VSMCs were removed from the paraformaldehyde and washed in PBS 3 times. Permeabilized with 0.1% Triton‐X, cells were washed another 3 times with PBS and then incubated with 5% BSA for 30 minutes. After that, cells were incubated with rabbit anti‐active *β*‐Catenin (CST) overnight at 4 °C, Alexa Fluor 488–labeled secondary antibodies (Beyotime) for 1 h at room temperature, and DAPI (Servicebio) for 5 min at room temperature. Following this, cells were washed three times with PBS. Imaging was performed using LSM 880 with Airyscan.

### Statistical Analyses

All statistical analyses were conducted using GraphPad Prism 8.0. Values were expressed as means ± SEM. Comparisons between the two groups were done using two‐tailed Student's *t*‐tests or nonparametric Mann–Whitney U test. Multiple comparisons were tested by ANOVA followed by Bonferroni's or Dunnett's post‐hoc analysis. Additionally, the correlation was confirmed using Spearman's rank correlation coefficient. *p* < 0.05 was considered statistically significant.

### Ethical Statement

All animal experiments abided by animal ethical standards and were approved by the Ethics Committee of Zhongshan School of Medicine, Sun Yat‐sen University (SYSU‐ACUC‐2022‐B0053). The clinical trial protocol and human samples collection were performed with the Declaration of Helsinki and approved by the Ethics Committee of The Eighth Affiliated Hospital of Sun Yat‐sen University (2023‐100‐01). All participants or legal representatives gave written informed consent before entering the study.

## Conflict of Interest

The authors declare no conflict of interest.

## Author Contributions

Z.Z., B.D., D.H., and J.M. contributed equally to this work. Z.Z., J.T., and H.H. conceived the research. Z.Z., B.D., D.H., and J.M. performed the major experiments. Y.K., H.Z., C.X., T.Y., X.Z., and Z.Z. contributed to the collection of clinical populations and blood samples. Z.H. and J.C. collected the arterial samples. Z.Z., A.H., and R.W. analyzed and interpreted the clinical data. Z.Z., Y.C., and H.H. drafted and revised the manuscript, with comments from all authors. H.Y. supervised English spelling. All authors approved the final version of the article.

## Supporting information



Supporting Information

## Data Availability

The data that support the findings of this study are available from the corresponding author upon reasonable request.

## References

[1] a) M. Vervloet , M. Cozzolino , Kidney Int. 2017, 91, 808; 27914706 10.1016/j.kint.2016.09.024

[2] K. Matsushita , S. H. Ballew , R. Kalyesubula , A. Y.‐M. Wang , E. Schaeffner , R. Agarwal , Nat. Rev., Nephrol. 2022, 18, 696. 36104509 10.1038/s41581-022-00616-6

[3] a) P. Lanzer , F. M. Hannan , J. D. Lanzer , J. Janzen , P. Raggi , D. Furniss , M. Schuchardt , R. Thakker , P.‐W. Fok , J. Saez‐Rodriguez , A. Millan , Y. Sato , R. Ferraresi , R. Virmani , C. St. Hilaire , J. Am. College Cardiol. 2021, 78, 1145; 10.1016/j.jacc.2021.06.049 PMC8439554 34503684

[4] a) X. Dai , S. Liu , L. Cheng , T. Huang , H. Guo , D. Wang , M. Xia , W. Ling , Y. Xiao , Circ. Res. 2022, 130, 1565; 35410483 10.1161/CIRCRESAHA.121.320251

[5] L. Ouyang , C. Yu , Z. Xie , X. Su , Z. Xu , P. Song , J. Li , H. Huang , Y. Ding , M. H. Zou , Circulation 2022, 145, 1784. 35582948 10.1161/CIRCULATIONAHA.121.057868 PMC9197997

[6] a) F. I. Fadel , M. F. Elshamaa , R. G. Essam , E. A. Elghoroury , G. S. M. El‐Saeed , S. E. El‐Toukhy , M. H. Ibrahim , J. Biomed. Sci. 2014, 10, 36; PMC3976446 24711748

[7] a) W. Durante , Nutrients 2019, 11, 2092;31487814

[8] B. Jiang , J. Zhang , G. Zhao , M. Liu , J. Hu , F. Lin , J. Wang , W. Zhao , H. Ma , C. Zhang , C. Wu , L. Yao , Q. Liu , X. Chen , Y. Cao , Y. Zheng , C. Zhang , A. Han , D. Lin , Q. Li , Mol. Cell 2022, 82, 1821. 35381197 10.1016/j.molcel.2022.03.016

[9] Y. Johmura , T. Yamanaka , S. Omori , T.‐W. Wang , Y. Sugiura , M. Matsumoto , N. Suzuki , S. Kumamoto , K. Yamaguchi , S. Hatakeyama , T. Takami , R. Yamaguchi , E. Shimizu , K. Ikeda , N. Okahashi , R. Mikawa , M. Suematsu , M. Arita , M. Sugimoto , K. I. Nakayama , Y. Furukawa , S. Imoto , M. Nakanishi , Science 2021, 371, 265. 33446552 10.1126/science.abb5916

[10] T. Bertero , W. M. Oldham , K. A. Cottrill , S. Pisano , R. R. Vanderpool , Q. Yu , J. Zhao , Y. Tai , Y. Tang , Y.‐Y. Zhang , S. Rehman , M. Sugahara , Z. Qi , J. Gorcsan , S. O. Vargas , R. Saggar , R. Saggar , W. D. Wallace , D. J. Ross , K. J. Haley , A. B. Waxman , V. N. Parikh , T. De Marco , P. Y. Hsue , A. Morris , M. A. Simon , K. A. Norris , C. Gaggioli , J. Loscalzo , J. Fessel , et al., J. Clin. Invest. 2016, 126, 3313. 27548520 10.1172/JCI86387 PMC5004943

[11] N. S. Rachedi , Y. Tang , Y.‐Y. Tai , J. Zhao , C. Chauvet , J. Grynblat , K. K. F. Akoumia , L. Estephan , S. Torrino , C. Sbai , A. Ait‐Mouffok , J. D. Latoche , Y. Al Aaraj , F. Brau , S. Abélanet , S. Clavel , Y. Zhang , C. Guillermier , N. V. G. Kumar , S. Tavakoli , O. Mercier , M. G. Risbano , Z.‐K. Yao , G. Yang , O. Ouerfelli , J. S. Lewis , D. Montani , M. Humbert , M. L. Steinhauser , C. J. Anderson , et al., Cell Metab. 2024, 36, 1335. 38701775 10.1016/j.cmet.2024.04.010 PMC11152997

[12] A. A. Gibb , E. K. Murray , A. T. Huynh , R. B. Gaspar , T. L. Ploesch , K. Bedi , A. A. Lombardi , P. K. Lorkiewicz , R. Roy , Z. Arany , D. P. Kelly , K. B. Margulies , B. G. Hill , J. W. Elrod , Circulation 2022, 145, 1625. 35605036 10.1161/CIRCULATIONAHA.121.057879 PMC9179236

[13] G. Zhong , S. Su , J. Li , H. Zhao , D. Hu , J. Chen , S. Li , Y. Lin , LimingWen, X. L. , G. Xian , D. Xu , Q. Zeng , Sci. Adv. 2023, 9, adg0478. 10.1126/sciadv.adg0478 PMC10413650 37267365

[14] a) S. Stegen , G. Rinaldi , S. Loopmans , I. Stockmans , K. Moermans , B. Thienpont , S.‐M. Fendt , P. Carmeliet , G. Carmeliet , Development Cell 2020, 53, 530; 10.1016/j.devcel.2020.05.001 32470321

[15] S. F. Traynelis , L. P. Wollmuth , C. J. McBain , F. S. Menniti , K. M. Vance , K. K. Ogden , K. B. Hansen , H. Yuan , S. J. Myers , R. Dingledine , D. Sibley , Pharmacol. Rev. 2010, 62, 405. 20716669 10.1124/pr.109.002451 PMC2964903

[16] A. D. Hogan‐Cann , C. M. Anderson , Trends Pharmacol. Sci. 2016, 37, 750. 27338838 10.1016/j.tips.2016.05.012

[17] I. Laketić‐ljubojević , L. J. Suva , F. J. M. Maathuis , D. Sanders , T. M. Skerry , Bone 1999, 25, 631. 10593407 10.1016/s8756-3282(99)00224-0

[18] C. Chenu , C. M. Serre , C. Raynal , B. Burt‐Pichat , P. D. Delmas , Bone 1998, 22, 295. 9556127 10.1016/s8756-3282(97)00295-0

[19] a) B. Thompson , D. A. Towler , Nat. Rev. Endocrinol. 2012, 8, 529; 22473330 10.1038/nrendo.2012.36 PMC3423589

[20] a) W. J. Nelson , R. Nusse , Science 2004, 303, 1483; 15001769 10.1126/science.1094291 PMC3372896

[21] T. Cai , D. Sun , Y. Duan , P. Wen , C. Dai , J. Yang , W. He , Exp. Cell Res. 2016, 345, 206. 27321958 10.1016/j.yexcr.2016.06.007

[22] J.‐S. Shao , S.‐L. Cheng , J. M. Pingsterhaus , N. Charlton‐Kachigian , A. P. Loewy , D. A. Towler , J. Clin. Invest. 2005, 115, 1210. 15841209 10.1172/JCI24140 PMC1077175

[23] N. Bansal , R. Katz , C. Robinson‐Cohen , M. C. Odden , L. Dalrymple , M. G. Shlipak , M. J. Sarnak , D. S. Siscovick , L. Zelnick , B. M. Psaty , B. Kestenbaum , A. Correa , M. Afkarian , B. Young , I. H. de Boer , JAMA Cardiology 2017, 2, 314. 28002548 10.1001/jamacardio.2016.4652 PMC5832350

[24] B. Lidgard , N. Bansal , L. R. Zelnick , A. Hoofnagle , J. Chen , D. Colaizzo , M. Dobre , K. T. Mills , A. C. Porter , S. E. Rosas , M. J. Sarnak , S. Seliger , J. Sondheimer , M. K. Tamura , K. Yaffe , B. Kestenbaum , J. Am. Soc. Nephrol. 2022, 33, 1391. 35444055 10.1681/ASN.2021111435 PMC9257801

[25] V. K. Ramanan , J. Graff‐Radford , J. Syrjanen , D. Shir , A. Algeciras‐Schimnich , J. Lucas , Y. A. Martens , M. M. Carrasquillo , G. S. Day , N. Ertekin‐Taner , C. Lachner , F. B. Willis , D. S. Knopman , C. R. Jack , R. C. Petersen , P. Vemuri , N. Graff‐Radford , M. M. Mielke , Neurology 2023, 101, e399. 37580163 10.1212/WNL.0000000000207675 PMC10573134

[26] D. Tzur Bitan , I. Krieger , A. Berkovitch , D. Comaneshter , A. Cohen , General Hospital Psychiatry 2019, 58, 1. 30807892 10.1016/j.genhosppsych.2019.01.007

[27] J. D. Mitchell , R. Paisley , P. Moon , E. Novak , T. C. Villines , JACC: Cardiovas. Imaging 2018, 11, 1799. 10.1016/j.jcmg.2017.09.003 29153576

[28] a) X. Li , A. Liu , C. Xie , Y. Chen , K. Zeng , C. Xie , Z. Zhang , P. Luo , H. Huang , Kidney Int. 2024, 105, 115; 37914087 10.1016/j.kint.2023.09.028

[29] A. L. Durham , M. Y. Speer , M. Scatena , C. M. Giachelli , C. M. Shanahan , Cardiovasc. Res. 2018, 114, 590. 29514202 10.1093/cvr/cvy010 PMC5852633

[30] a) M. Mercado‐Gomez , N. Goikoetxea‐Usandizaga , A. J. C. Kerbert , L. U. Gracianteparaluceta , M. Serrano‐Macia , S. Lachiondo‐Ortega , R. Rodriguez‐Agudo , C. Gil‐Pitarch , J. Simon , I. Gonzalez‐Recio , M. F. Fondevila , P. Santamarina‐Ojeda , M. F. Fraga , R. Nogueiras , J. L. Heras , R. Jalan , M. L. Martinez‐Chantar , T. C. Delgado , Metabolism 2024, 158, 155952; 38906371 10.1016/j.metabol.2024.155952

[31] A. Reiner , J. Levitz , Neuron 2018, 98, 1080. 29953871 10.1016/j.neuron.2018.05.018 PMC6484838

[32] K. Abe , M. Takeichi , Neuron 2007, 53, 387. 17270735 10.1016/j.neuron.2007.01.016

[33] Y. Xu , H. Saini , M. Zhang , V. Elimban , N. Dhalla , Cardiovasc. Res. 2006, 72, 163. 16901476 10.1016/j.cardiores.2006.06.028

[34] a) Q. Ding , W. Xia , J.‐C. Liu , J.‐Y. Yang , D.‐F. Lee , J. Xia , G. Bartholomeusz , Y. Li , Y. Pan , Z. Li , R. C. Bargou , J. Qin , C.‐C. Lai , F.‐J. Tsai , C.‐H. Tsai , M.‐C. Hung , Mol. Cell 2005, 19, 159; 16039586 10.1016/j.molcel.2005.06.009

[35] A. l. Bedel , A. Nègre‐Salvayre , S. Heeneman , M.‐H. Grazide , J.‐C. Thiers , R. Salvayre , F. Maupas‐Schwalm , Circ. Res. 2008, 103, 694. 18703780 10.1161/CIRCRESAHA.107.166405

[36] C. Yang , B. Ko , C. T. Hensley , L. Jiang , A. T. Wasti , J. Kim , J. Sudderth , M. A. Calvaruso , L. Lumata , M. Mitsche , J. Rutter , M. E. Merritt , R. J. DeBerardinis , Mol. Cell 2014, 56, 414. 25458842 10.1016/j.molcel.2014.09.025 PMC4268166

[37] Y. Yu , H. Newman , L. Shen , D. Sharma , G. Hu , A. J. Mirando , H. Zhang , E. Knudsen , G.‐F. Zhang , M. J. Hilton , C. M. Karner , Cell Metab. 2019, 29, 966. 30773468 10.1016/j.cmet.2019.01.016 PMC7062112

[38] T. Huang , R. Liu , X. Fu , D. Yao , M. Yang , Q. Liu , W. W. Lu , C. Wu , M. Guan , Stem Cells 2017, 35, 411. 27501743 10.1002/stem.2470

[39] B. J. Altman , Z. E. Stine , C. V. Dang , Nat. Rev., Cancer 2016, 16, 619. 27492215 10.1038/nrc.2016.71 PMC5484415

[40] X. Xia , G. Cao , G. Sun , L. Zhu , Y. Tian , Y. Song , C. Guo , X. Wang , J. Zhong , W. Zhou , P. Li , H. Zhang , J. Hao , Z. Li , L. Deng , Z. Yin , Y. Gao , J. Clin. Invest. 2020, 130, 5180. 32831293 10.1172/JCI129269 PMC7524468

[41] P. Paoletti , C. Bellone , Q. Zhou , Nat. Rev. Neurosci. 2013, 14, 383. 23686171 10.1038/nrn3504

[42] S. J. Dumas , G. Bru‐Mercier , A. Courboulin , M. Quatredeniers , C. Rucker‐Martin , F. Antigny , M. K. Nakhleh , B. Ranchoux , E. Gouadon , M. C. Vinhas , M. Vocelle , N. Raymond , P. Dorfmuller , E. Fadel , F. Perros , M. Humbert , S. Cohen‐Kaminsky , Circulation 2018, 137, 2371. 29444988 10.1161/CIRCULATIONAHA.117.029930

[43] T. K. Chen , D. H. Knicely , M. E. Grams , JAMA, J. Am. Med. Assoc. 2019, 322, 1294. 10.1001/jama.2019.14745 PMC7015670 31573641

[44] J. J. Carr , J. C. Nelson , N. D. Wong , M. McNitt‐Gray , Y. Arad , D. R. J. Jr , S. Sidney , D. E. Bild , O. D. Williams , R. C. Detrano , Radiology 2005, 234, 35. 15618373 10.1148/radiol.2341040439

[45] L. Ouyang , X. Su , W. Li , L. Tang , M. Zhang , Y. Zhu , C. Xie , P. Zhang , J. Chen , H. Huang , J. Clin. Invest. 2021, 131, e146985. 34003800 10.1172/JCI146985 PMC8279589

