# Supplementary material for: GLS1‐Mediated Redundancy in Glutamate Accelerates Arterial Calcification via Activating NMDAR/Ca2+/β‐Catenin Pathway
Source: Adv Sci (Weinh). 2025 Apr 28;12(21):2414252. doi: 10.1002/advs.202414252 (PMC12140355; doi:10.1002/advs.202414252)
Supplement: Supplementary file 1 — Supporting Information [file ADVS-12-2414252-s001.pdf]

ADVANCED SCIENCE
Open Access

Supporting Information

for *Adv. Sci.*, DOI 10.1002/advs.202414252

GLS1-Mediated Redundancy in Glutamate Accelerates Arterial Calcification via Activating NMDAR/Ca^{2+}/β-Catenin Pathway

Ziting Zhou, Bing Dong, Dayu He, Jianshuai Ma, Yun Kong, Huijin Zhu, Chen Xie, Tiecheng Yang, Xin Zhen, Zhengzhipeng Zhang, Zhaohui He, Jinkun Cheng, Aoran Huang, Jie Chen, Ruo Wu, Huiyong Yin, Yanlian Chen*, Jun Tao* and Hui Huang**

Supporting Information

GLS1-Mediated Redundancy in Glutamate Accelerates Arterial Calcification via Activating NMDAR/Ca^{2+}/β-Catenin Pathway

Ziting Zhou[1], Bing Dong[1], Dayu He[1], Jianshuai Ma[1], Yun Kong[1], Huijin Zhu[1], Chen Xie[2], Tiecheng Yang[3], Xin Zhen[3], Zhengzhipeng Zhang[1], Chaohui He[4], Jinkun Cheng[1], Aoran Huang[1], Jie Chen[5], Ruo Wu[1], Huiyong Yin[6], Yanlian Chen[2*], Jun Tao[7*], and Hui Huang[1*].*

List of Supporting Information

Supplemental Figures and Legends

1) **Figure S1.** Glutamate metabolism is related to arterial calcification.

2) **Figure S2.** Depression is associated with arterial calcification in CKD patients.

3) **Figure S3.** Increased GLS1 expression is observed in calcified arteries.

4) **Figure S4.** The efficiency of GLS1 knockdown or overexpression and the microscopic images of Alizarin Red staining with indicated treatments.

5) **Figure S5.** Glutamate aggravates osteogenic reprogramming of VSMCs by activating and upregulating NMDAR.

6) **Figure S6.** ERK is the major downstream molecule of glutamate-NMDAR axis.

7) **Figure S7.** Glutamate-activated NMDAR induces nuclear translocation of *β*-Catenin to promote osteogenic transdifferentiation of VSMCs.

8) **Figure S8.** Glutamate-NMDAR axis activates *β*-Catenin to exacerbate osteogenic transdifferentiation of VSMCs.

9) **Figure S9.** ERK inhibitors hinder the activation of *β*-Catenin.

10) **Figure S10.** *β*-Catenin is the main molecule in the development of GLS1-induced osteogenic transdifferentiation of VSMCs.

Supplemental Tables

1) **Supplementary Table S1.** Baseline characteristics in healthy and CKD patients.

2) **Supplementary Table S2.** The Relative siRNA in this work.

3) **Supplementary Table S3.** Used antibodies in this work.

4) **Supplementary Table S4.** Sequence of primers for q-PCR.

Supplemental Figures and Legend:

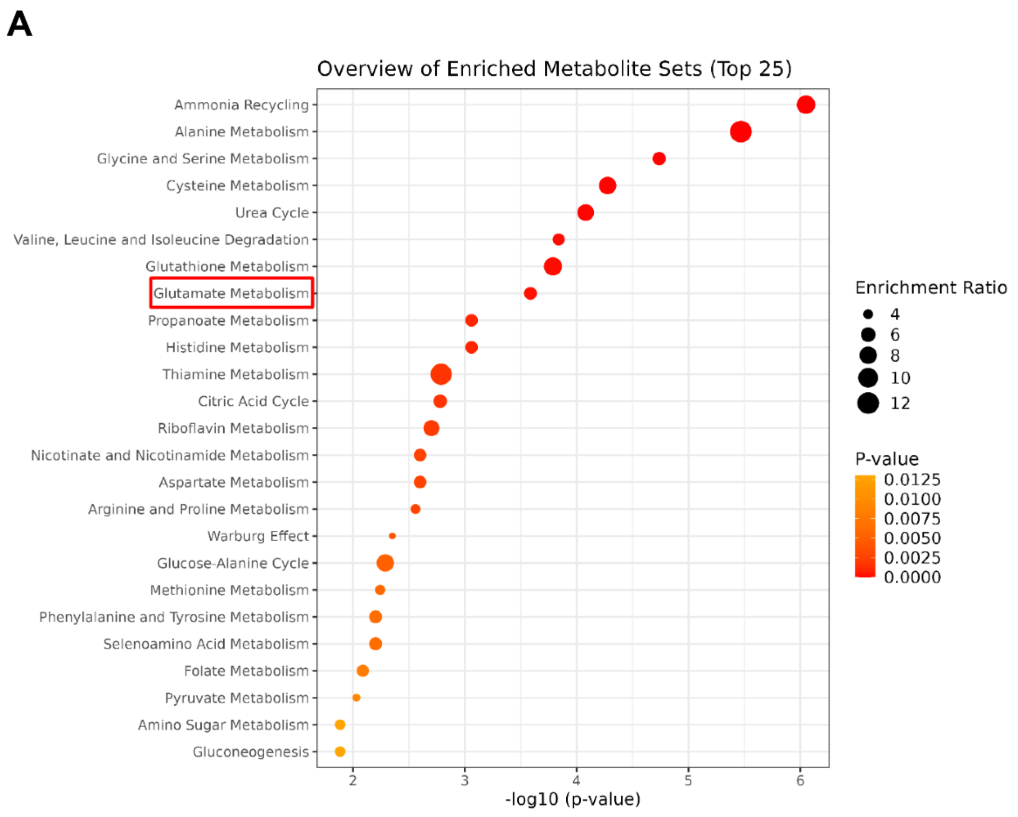

Figure S1. Glutamate metabolism is related to arterial calcification. A) KEGG pathway enrichment analysis using the MetaboAnalyst database revealed that blood metabolites causally associated with arterial calcification involved in glutamate metabolism.

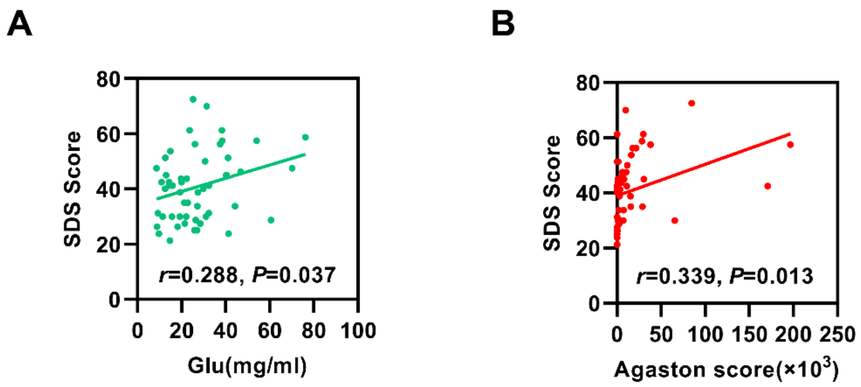

Figure S2. Depression is associated with arterial calcification in CKD patients. A) The relationship between Self-Rating Depression Scale (SDS Score) and glutamate (Glu) was analyzed (n=53). B) Correlation analysis between vascular calcification and SDS score was conducted (n=53). Spearman's rank correlation coefficient was used.

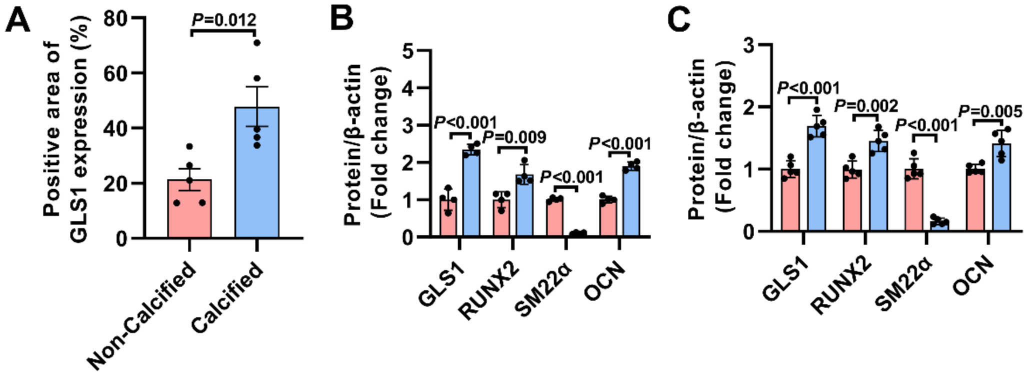

Figure S3. Increased GLS1 expression is observed in calcified arteries. A) Statistical diagram of immunohistochemical images of GLS1 staining in patients' arteries with or without calcification (n=5). B) The expression of GLS1 protein was confirmed from mice aortas in either the control or $VitD_3$-induced arterial calcification groups (n=4). C) The levels of GLS1 protein from mice aortas in control or CKD-related arterial calcification groups were shown by statistical diagram of representative immunoblots (n=5). Data are shown as means ± SEM. Two-tailed, unpaired Student's t test was used in (A); One-way analysis of variance (ANOVA) with Bonferroni's test was used in (B) and (C).

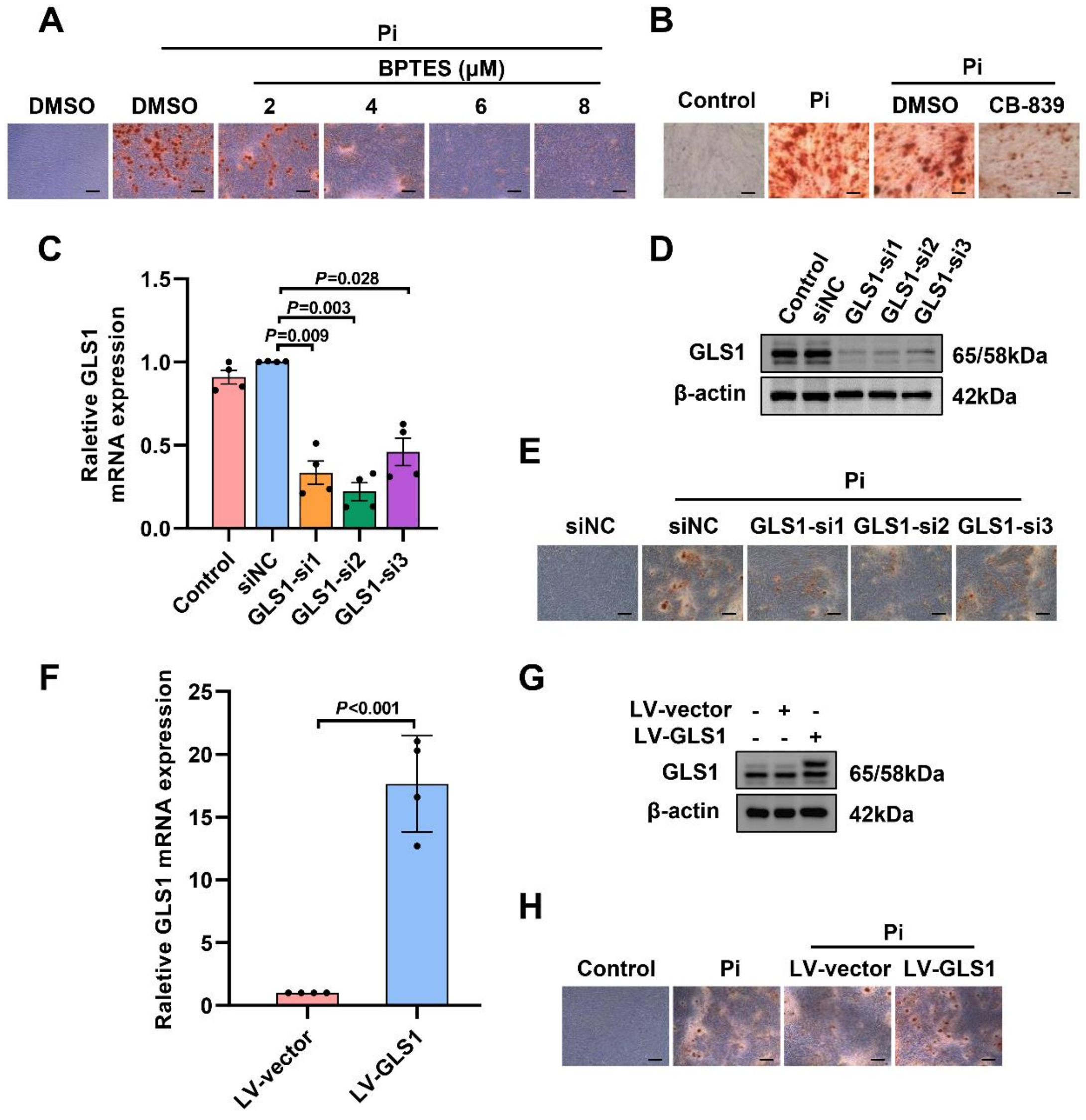

Figure S4. The efficiency of GLS1 knockdown or overexpression and the microscopic images of Alizarin Red staining with indicated treatments. A) Representative microscopic images of Alizarin Red staining from VSMCs incubated with Pi and GLS1 inhibitor (BPTES) for 7 days (n=3). Scale bar: 200μm. B) VSMCs treated with CB-839 (1μM) were stained for mineralization by the microscopic images of Alizarin Red staining (n=3). Scale bar: 200μm. C-D) The efficiency GLS1-siRNA knockdown was verified by the change of mRNA and protein levels (n=4). E) Pi-mediated osteogenic transdifferentiation of VSMCs pretransfected with siNC or GLS1-siRNA were observed through the microscopic images of Alizarin Red staining (n=3). F-G) mRNA

and protein of the efficiency of LV-GLS1 overexpression (*n*=4). H) Representative microscopic images of Alizarin Red staining in Pi triggered osteogenic transdifferentiation of VSMCs preinfected with LV-vector or LV-GLS1 (*n*=3). Scale bar: 200μm. All values are expressed as means ± SEM. One-way ANOVA with Bonferroni's test was used in (C); Two-tailed, unpaired Student's t test was used in (F).

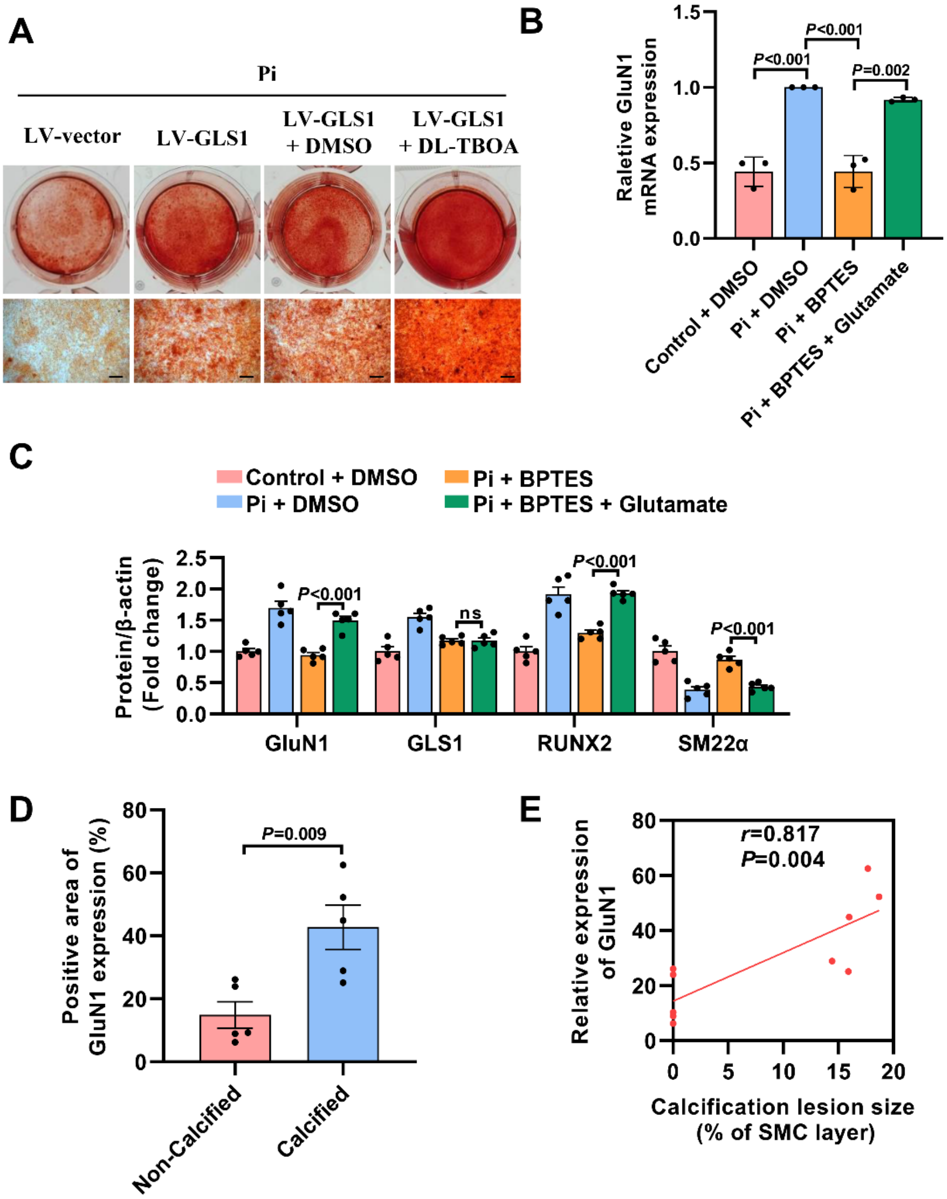

Figure S5. Glutamate aggravates osteogenic reprogramming of VSMCs by activating and upregulating NMDAR. A) The whole-well images and the corresponding microscopic views of Alizarin Red staining was derived from VSMCs incubated with Pi and overexpression of GLS1 in the absence or presence of DL-TBOA (glutamate transport inhibitor) (*n*=4). Scale bar: 200μm. B) mRNA level of GluN1 was tested by quantitative PCR from VSMCs in the indicated group (*n*=3). C) The statistical diagram of GluN1 protein levels from VSMCs in the indicated group were measured by western

blot assay (*n*=5). D) Statistical diagram of representative immunohistochemical images of GLS1 staining in patients' arteries with or without calcification (*n*=5). E) Spearman's correlation analysis for relative expression of GluN1 correlated with calcification lesion size (% of SMC layer) in patients with or without calcification (*n*=5). Data are shown as means ± SEM. One-way ANOVA with Dunnett's test was used in (B) and (C); Two-tailed, unpaired Student's t test was used in (D); Spearman's rank correlation coefficient was used.

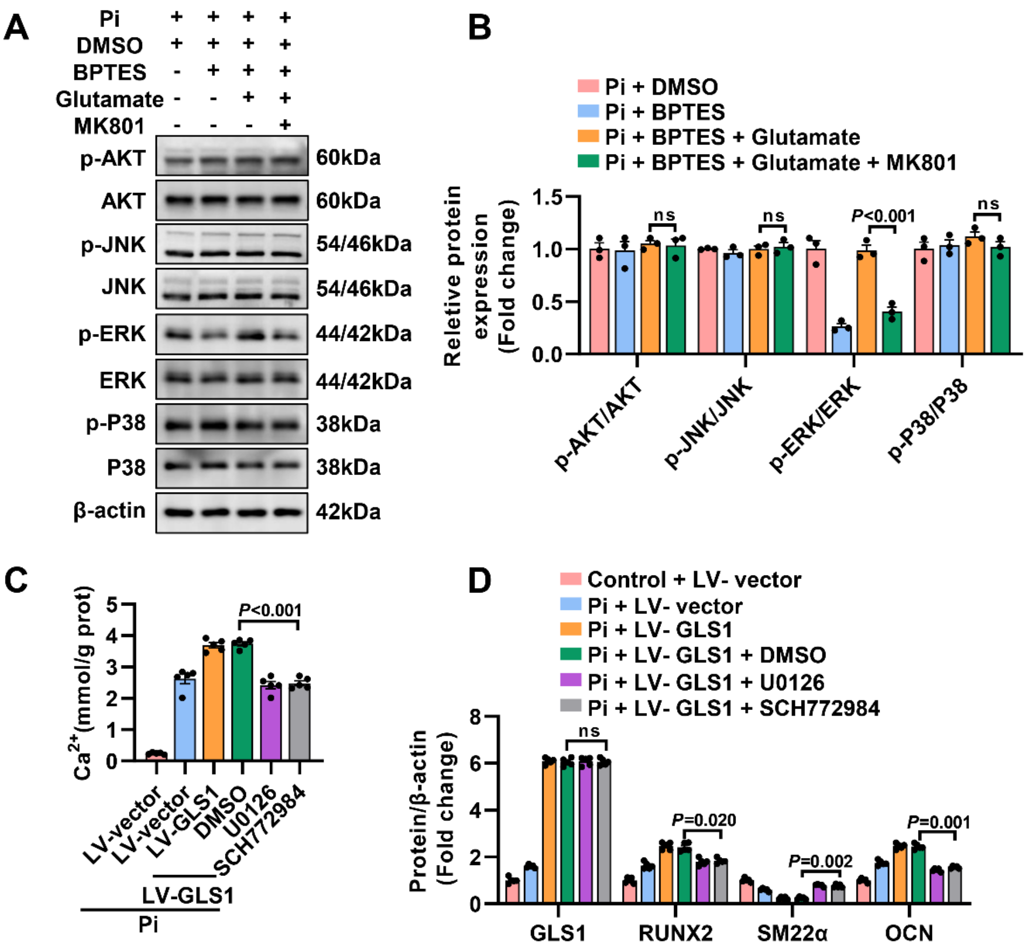

Figure S6. ERK is the major downstream molecule of glutamate-NMDAR axis. A) Representative immunoblotting depicted the levels of p-AKT, AKT, p-JNK (phosphate-c-Jun N-terminal kinase), JNK, p-ERK, ERK, p-P38 and P38 proteins following the indicated treatment (*n*=3). B) Statistical diagram exhibited the changes of p-AKT, p-JNK, p-ERK and p-P38 levels, which were normalized to their corresponding total protein intensities. C) Quantitative analysis of calcium content was performed following the indicated treatments (*n*=5). D) Statistical diagram showed the effects of ERK inhibitors (U0126 and SCH772984) on osteogenic transdifferentiation of VSMCs incubated with Pi and GLS1 overexpression (*n*=5). All values are expressed as means ± SEM. One-way ANOVA with Dunnett's test was used in (B) and (C), or Bonferroni's test was used in (D).

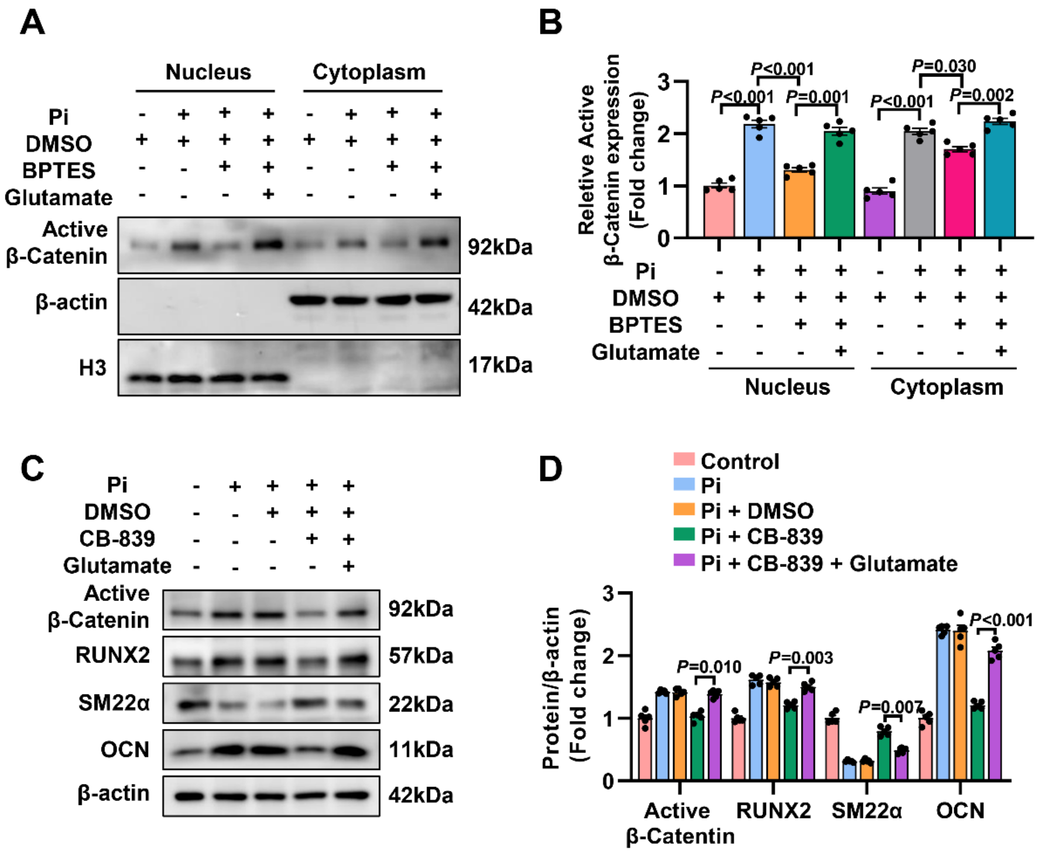

Figure S7. Glutamate-activated NMDAR induces nuclear translocation of *β*-Catenin to promote osteogenic transdifferentiation of VSMCs. A) The expression of active *β*-Catenin protein levels from the nucleus, cytoplasm of VSMCs in the indicated group were measured by western blot assay (n=5). B) The statistical analysis showed the levels of active *β*-Catenin in the nucleus (normalized to H3) and cytoplasm (normalized to β-actin) (n=5). C-D) Western Blots and statistical diagram showed the changes of active *β*-Catenin levels and markers of osteogenic reprogramming (all normalized to *β*-actin) in the indicated groups (n=5). Data are shown as means ± SEM. One-way ANOVA with Bonferroni's test was used in (B), or Dunnett's test was used in (D).

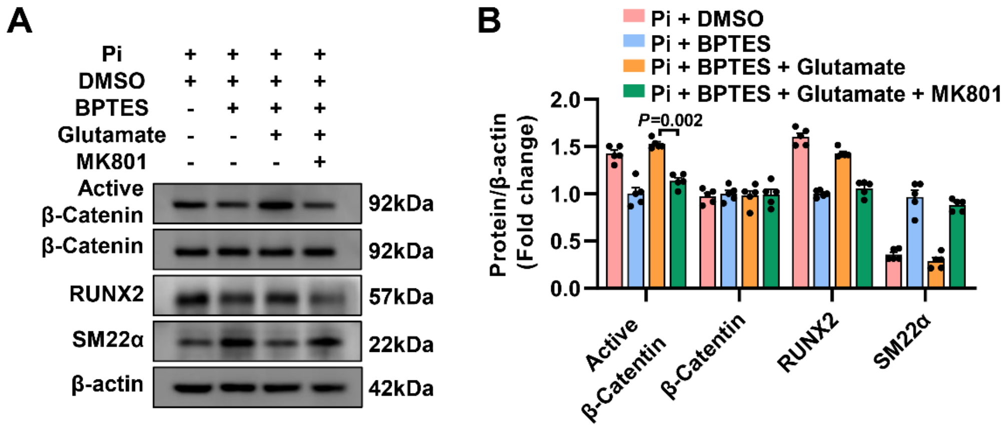

Figure S8. Glutamate-NMDAR axis activates β-Catenin to exacerbate osteogenic transdifferentiation of VSMCs. A-B) The expressions of active β-Catenin protein in VSMCs with the indicated treatment were evaluated by Western Blotting assay (n=5). All values are expressed as means ± SEM. One-way ANOVA with Bonferroni's test was used in (B).

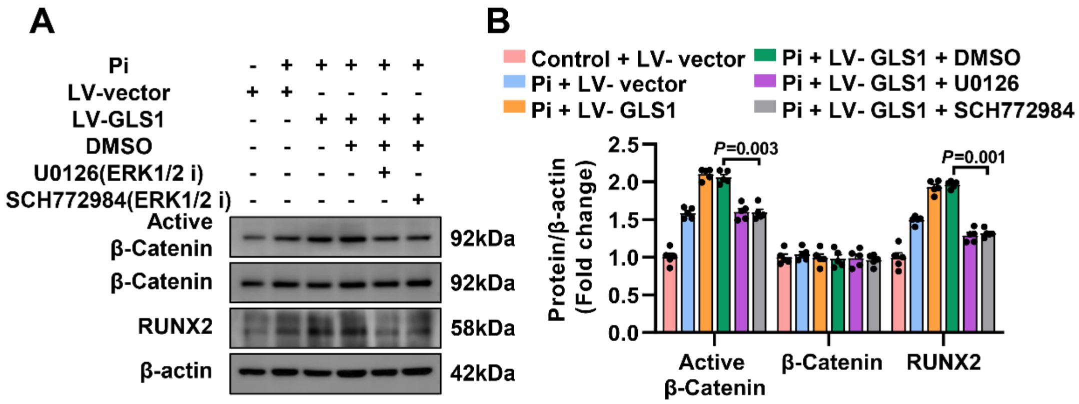

Figure S9. ERK inhibitors hinder the activation of *β*-Catenin. A-B) After overexpression of GLS1, western blot analysis of active *β*-Catenin protein expression in VSMCs incubated with Pi in the absence or presence of ERK inhibitors (n=5). Data are shown as means ± SEM. One-way ANOVA with Bonferroni's test was used in (B).

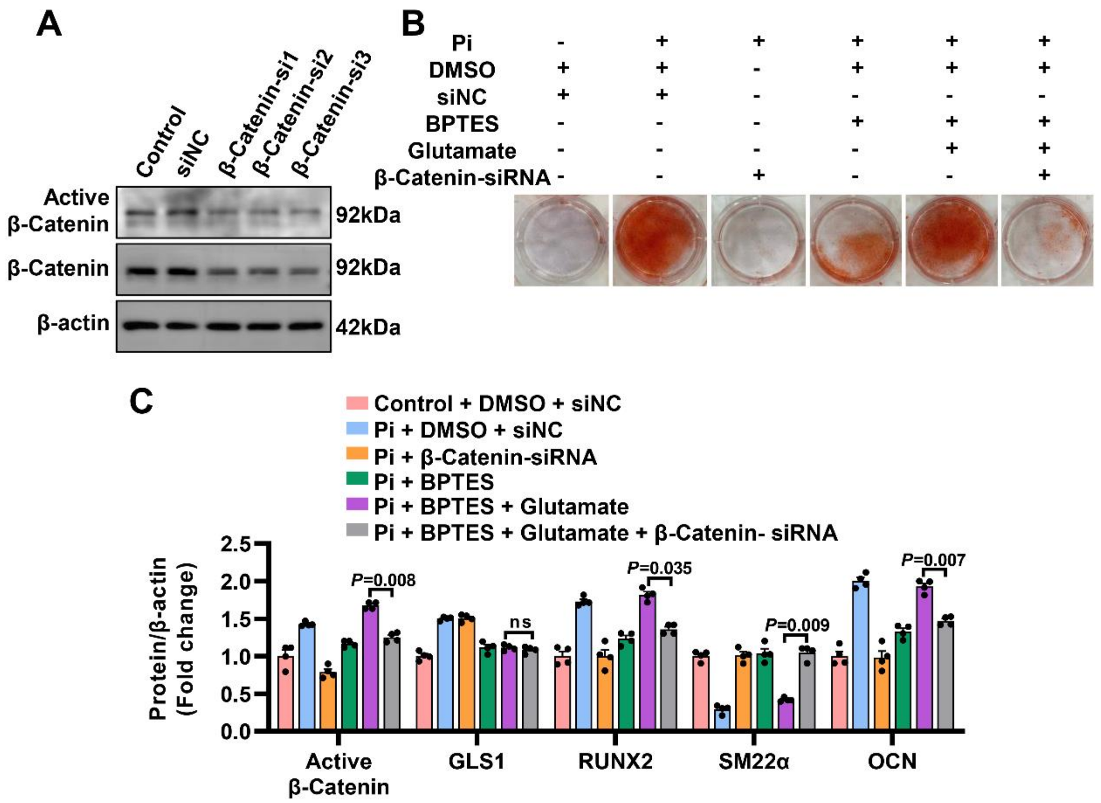

Figure S10. *β*-Catenin is the main molecule in the development of GLS1-induced osteogenic transdifferentiation of VSMCs. A) Representative immunoblots of *β*-Catenin-siRNA knockdown efficiency (*n*=3). B) Representative images of Alizarin Red staining in VSMCs with the indicated treatment (*n*=3). C) Representative immunoblotting of active *β*-Catenin protein levels with indicated treatment were depicted by statistical diagram (*n*=4). All values are expressed as means ± SEM. One-way ANOVA with Bonferroni's test was used in (C).

Table S1: Baseline characteristics in healthy controls and CKD patients

**Variables**	**Healthy controls (*n*=58)**	**CKD (*n*=65)**	***P* Value**
Age [years]	50.26±10.68	53.77±13.22	0.111
male [n %]	30 (44.8%)	36 (55.4%)	0.720
SBP [mmHg]	120.00 (115.00-132.00)	142.00 (128.50-162.00)	<0.001
DBP [mmHg]	77.00 (66.00-83.00)	88.00 (73.00-102.00)	<0.001
CREA [μmol/L]	59.12 (53.59-72.42)	940.66 (630.23-1174.95)	<0.001
BUN [mmol/L]	4.67 (3.90-5.68)	23.20 (16.98-31.27)	<0.001
TC [mmol/L]	4.76 (4.21-5.42)	3.90 (3.02-4.74)	<0.001
TG [mmol/L]	1.49 (1.0-1.98)	1.43 (0.9-1.89)	0.287
LDL-C [mmol/L]	2.71 ± 0.91	2.46 ± 0.94	0.134
HDL-C [mmol/L]	1.38 ± 0.35	1.03 ± 0.31	<0.001
Ca [mmol/L]	2.22 ± 0.10	2.23 ± 0.19	0.863
Pi [mmol/L]	1.19 (1.06-1.36)	1.95 (1.51-2.43)	<0.001
GLU [mmol/L]	4.78 (4.1-5.30)	5.03 (4.41-5.66)	0.063

Values are expressed as mean ± SD, median (interquartile range), or number (%). Statistical significance was assessed using one-way ANOVA followed by Bonferroni test. CKD, chronic kidney disease; SBP, systolic blood pressure; DBP, diastolic blood pressure; CREA, creatinine; BUN, blood urea nitrogen; TC, total cholesterol; TG, triglyceride; LDL-C, low-density lipoprotein cholesterol; HDL-C, high-density lipoprotein cholesterol; Ca, calcium; Pi, inorganic phosphate; GLU, glucose.

Table S2. The Relative siRNA in this work.

**Name**	**Species**	**Sequences**
GLS1-siRNA-1	Human	UGAAUAAGAUGGCUGGUAA
GLS1-siRNA-2	Human	GAAAGUGGAGAUCGAAAUU
GLS1-siRNA-3	Human	CGAAAUACAUUGAGUUUGA
β-Catenin-siRNA-1	Human	CCAUGGAACCAGACAGAAA
β-Catenin-siRNA-2	Human	CCACUAAUGUCCAGCGUUU
β-Catenin-siRNA-3	Human	AGAAAUAGUUGAAGGUUGU
Scramble-siRNA (siNC)	Human	UUCUCCGAACGUGUCACGU

Table S3. Used antibodies in this work.

The antibodies used for Western Blotting			
**Antibody**	**Company**	**Identifiers**	**Concentrations**
GLS1 Polyclonal antibody	Proteintech	12855-1-AP	1:1000
GLS1 Monoclonal antibody	Proteintech	66265-1-Ig	1:1000
RUNX2 antibody	Proteintech	20700-1-AP	1:1000
RUNX2 antibody	Abcam	ab236639	1:1000
RUNX2 antibody	ABclonal	A11753	1:1000
BMP2 antibody	Proteintech	66383-1-Ig	1:1000
SM22α antibody	Proteintech	10493-1-AP	1:1000
GluN1 antibody	Proteintech	27676-1-AP	1:1000
P-ERK	Cell Signaling Technology	4370S	1:1000
ERK	Cell Signaling Technology	4695S	1:1000
P-P38	Proteintech	28796-1-AP	1:1000
P38	Proteintech	14064-1-AP	1:1000
P-JNK	Cell Signaling Technology	4668S	1:1000
JNK	Cell Signaling Technology	9252S	1:1000
P-AKT	Proteintech	66444-1-IG	1:1000
AKT	Proteintech	10176-2-AP	1:1000

(Active) *β*-Catenin antibody	Cell Signaling Technology	8814S	1:1000
β-Catenin antibody	Proteintech	10493-1-AP	1:1000
OCN	Abclonal	A20800	1:1000
Histone H3	Proteintech	17168-1-AP	1:1000
β-actin	Proteintech	66009-1-Ig	1:50000

The antibodies used for Immunohistochemistry/Immunofluorescence

**Antibody**	**Company**	**Identifiers**	**Concentrations**
GLS1 Monoclonal antibody	Proteintech	66265-1-Ig	1:400
SM22α antibody	Proteintech	10493-1-AP	1:400
NMDAR1 Antibody	SAB	SEM49488	1:100
(Active) *β*-Catenin antibody	Cell Signaling Technology	8814S	1:800

Table S4. Sequence of primers for q-PCR

**Genes**	**Species**	**Sequence (5'-3')**
GLS1-Forward	Human	GCTGCAGAGGGTCATGTTGA
GLS1-Reverse	Human	CTGTCCTTGGGGAAAGGGTT
GluN1-Forward	Human	TCGAGATTGCCTACAAGCGG
GluN1-Reverse	Human	GGATGGTACTGCTGCAGGTT
ACTB-Forward	Human	GGGCATGGGTCAGAAGGATT
ACTB-Reverse	Human	TCGATGGGGTACTTCAGGGT
GLS1-Forward	Mouse	TTTGCCGCATACACTGGAGA
GLS1-Reverse	Mouse	CATGGAGGGCTGTTCTGGAG
ACTB-Forward	Mouse	ATCGCTGCGCTGGTCG
ACTB-Reverse	Mouse	AACCGCTCGTTGCCAATAGT
